# Systematic Characterization of Dynamic Parameters of Intracellular Calcium Signals

**DOI:** 10.3389/fphys.2016.00525

**Published:** 2016-11-10

**Authors:** Laurent Mackay, Nicholas Mikolajewicz, Svetlana V. Komarova, Anmar Khadra

**Affiliations:** ^1^Department of Physiology, McGill UniversityMontreal, QC, Canada; ^2^Faculty of Dentistry, McGill UniversityMontreal, QC, Canada; ^3^Shriners Hospital for Children-CanadaMontreal, QC, Canada

**Keywords:** algorithm, calcium imaging, kinetics, osteoclast pathophysiology, parameter characterization, purinergic/P2 receptors, real-time imaging

## Abstract

Dynamic processes, such as intracellular calcium signaling, are hallmark of cellular biology. As real-time imaging modalities become widespread, a need for analytical tools to reliably characterize time-series data without prior knowledge of the nature of the recordings becomes more pressing. The goal of this study is to develop a signal-processing algorithm for MATLAB that autonomously computes the parameters characterizing prominent single transient responses (TR) and/or multi-peaks responses (MPR). The algorithm corrects for signal contamination and decomposes experimental recordings into contributions from drift, TRs, and MPRs. It subsequently provides numerical estimates for the following parameters: time of onset after stimulus application, activation time (time for signal to increase from 10 to 90% of peak), and amplitude of response. It also provides characterization of the (i) TRs by quantifying their area under the curve (AUC), response duration (time between 1/2 amplitude on ascent and descent of the transient), and decay constant of the exponential decay region of the deactivation phase of the response, and (ii) MPRs by quantifying the number of peaks, mean peak magnitude, mean periodicity, standard deviation of periodicity, oscillatory persistence (time between first and last discernable peak), and duty cycle (fraction of period during which system is active) for all the peaks in the signal, as well as coherent oscillations (i.e., deterministic spikes). We demonstrate that the signal detection performance of this algorithm is in agreement with user-mediated detection and that parameter estimates obtained manually and algorithmically are correlated. We then apply this algorithm to study how metabolic acidosis affects purinergic (P2) receptor-mediated calcium signaling in osteoclast precursor cells. Our results reveal that acidosis significantly attenuates the amplitude and AUC calcium responses at high ATP concentrations. Collectively, our data validated this algorithm as a general framework for comprehensively analyzing dynamic time-series.

## Introduction

**Figure 1:**
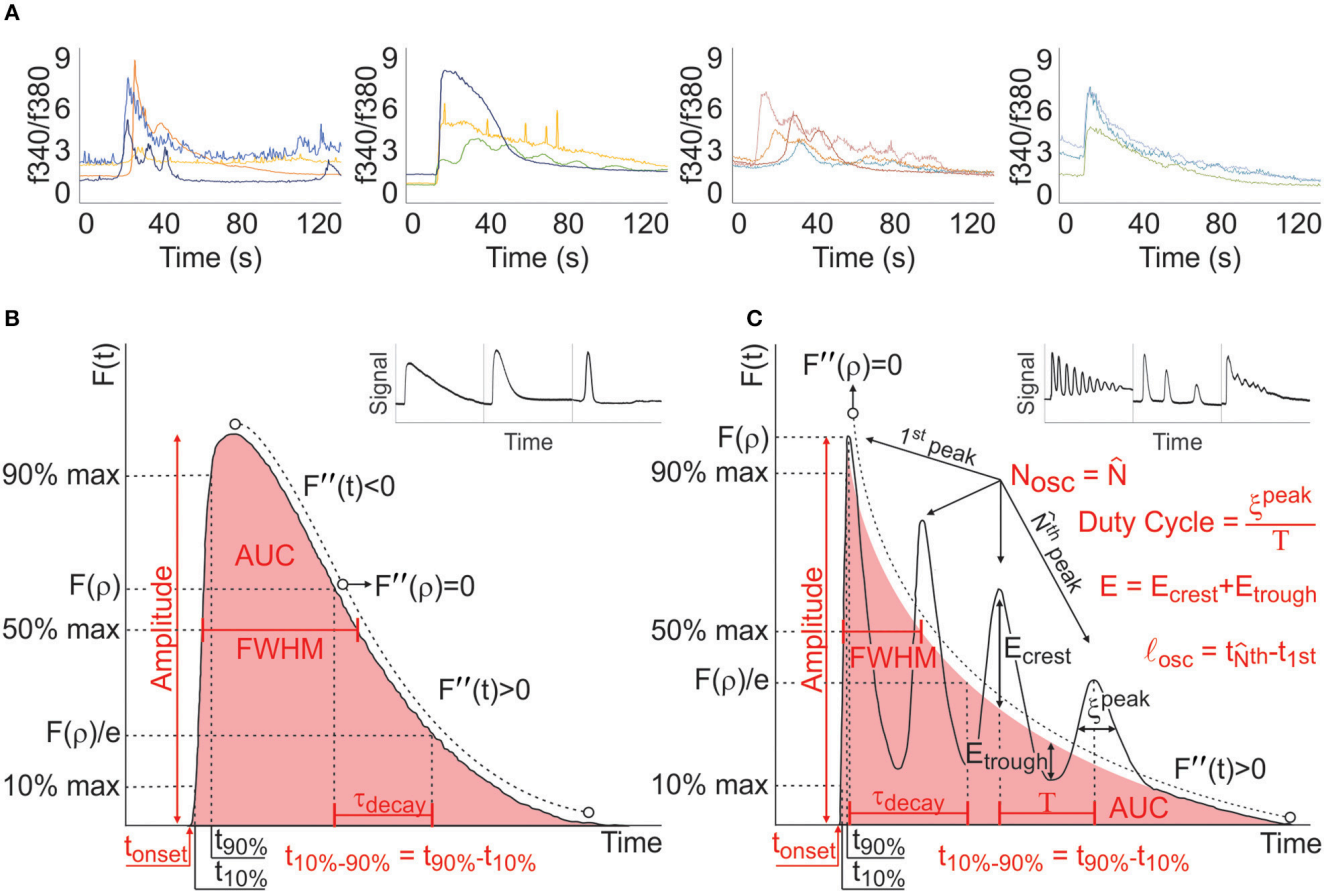
**Characterization of dynamic calcium signals**. **(A)** Examples of heterogeneity of [Ca^2+^]_i_ responses observed in various live-cell recordings; note many signature forms that TRs and MPRs can exhibit. **(B)** Analysis of parameters for single-peak TRs. Inset: Representative single-peak TRs. **(C)** Analysis of parameters for MPRs. Inset: Representative MPRs. Time of onset, t_onset_; area under curve, AUC; full-width half-max, FWHM; activation time, t_10–90%_; decay constant, τ_decay_; periodicity, T; number of peaks, N_osc_; oscillatory peak magnitude, E; width of oscillatory peaks, ξ^peak^; point of inflection in deactivation phase of TR, *F*″(ρ). Arrows/text in red illustrate how parameters of interest are obtained.

Cellular biology is vastly populated with dynamic processes, which can be altered dramatically or subtly by pathological causes. Calcium signals, characterized by fast and transient increases in cytosolic free calcium concentration ([Ca^2+^]_i_), which vary in amplitude and duration and can exhibit oscillatory dynamics with frequency-dependent downstream effects (Clapham, 2007), represent a prominent example of such dynamic processes (Figure 1A). To fully understand the data of such dynamic complexity, a robust methodology to analyse, and characterize these responses is necessary. Numerous studies have investigated [Ca^2+^]_i_ dynamics, but the analysis have in many cases been limited to qualitative assessments (Cao et al., 1997; Frame and de Feijter, 1997; Jorgensen et al., 1997; Jørgensen et al., 2003; Isakson et al., 2001; Romanello and D'Andrea, 2001). Studies that have pursued quantitative analysis of calcium time-series reported a number of different, often non-overlapping characteristics of the response (Table 1). In cases where experiments were conducted on a smaller-scale, manual analysis was achievable. However, to achieve larger-scale analyses for experiments with hundreds of individual recordings, open-source signal-processing algorithms are required and becoming increasingly relied on to overcome these bottlenecks in productivity. None of 11 published algorithms we examined provided a comprehensive analysis of the entire response observed within a recording (Table 2). As a direct consequence of the lack of a standardized methodology to quantify such data-sets, findings from various studies are challenging to compare, relate, and generalize. Hence, the motivation of this study was to achieve faster analysis while standardizing the methodology involved, thereby minimizing user-bias, and ensuring consistency in the analysis of complex physiological signals. While such a tool may or may not change the conclusions of individual studies, it would improve comparability between different studies, and potentially enable meta-analysis of different experiments. We have therefore developed an algorithm that addresses these concerns and focused on the dynamic signals generated by purinergic (P2) receptors to demonstrate its utility.

**Table 1:** **Commonly reported parameters in studies investigating calcium dynamics**.

**Amplitude**	**t_onset_**	**t_10−90%_**	**FWHM**	**AUC**	**τ_decay_**	**Period**	**References**
X	X						Abu Khamidakh et al., 2013
X			X			X	Appleby et al., 2015
X							Churchill et al., 1996
X	X					X	Dickinson and Parker, 2013
X			X				Francis et al., 2016
X	X						Hansen et al., 1993
X	X	X		X		X	James et al., 2011
X							Rast et al., 2015
X							Shabir and Southgate, 2008
X		X	X		X		Smith et al., 2009
X			X				Sun et al., 1997
X							Zhao et al., 2008

**Table 2:** **Published signal-processing algorithms**.

**Amplitude**	**t_onset_**	**t_10−−90%_**	**FWHM**	**AUC**	**τ_decay_**	**t_90−10*%*_**	**Period**	Classifier^*^	**References**
X		X	X						Bray et al., 2007
X		X				X			Ellefsen et al., 2014
	X					X			Fritzsche et al., 2015
								X	Juhola et al., 2015
X		X	X			X			Lock et al., 2015
X		X				X	X		Patel et al., 2015
X		X	X		X				Picht et al., 2007
							X		Ruffinatti et al., 2011
X		X				X			Stoehr et al., 2014
X			X		X		X		Steele and Steele, 2014
X									Wong et al., 2010

* Classifier: grouping of time-series by characteristic signature of response.

Purinergic receptors that evoke intracellular responses upon extracellular stimulation with nucleotides, such as ATP and ADP, are known to induce complex [Ca^2+^]_i_ signals. P2 receptors are subdivided into two families, P2X and P2Y receptors, which are omnipresent in virtually all mammalian tissue (Burnstock and Verkhratsky, 2009). The mammalian P2X receptor family, consisting of seven subtypes (P2X_1–7_), are ionotropic ligand-gated cation channels that can permit the influx of extracellular calcium upon stimulation (Kaczmarek-Hajek et al., 2012). The mammalian P2Y receptor family, consisting of eight subtypes (P2Y_1–2,4,6,11–14_), are metabotropic G-protein coupled receptors that can indirectly modulate the release of calcium from intracellular calcium stores through inositol triphosphate (von Kugelgen and Hoffmann, 2016). P2 receptors have been demonstrated to play an important role on bone physiology (Lenertz et al., 2015). Since individual bone cells commonly express multiple active P2 receptors (Gallagher and Buckley, 2002), responses to purinergic stimulation result in complex, concentration-dependent [Ca^2+^]_i_ transients (Xing et al., 2016). While it remains difficult to experimentally isolate the contribution of individual receptors, a number of studies have demonstrated that various P2 receptor subtypes have distinct calcium response kinetics and signatures. For instance, various P2X receptors desensitize at distinct rates under sustained agonist stimulation (Koshimizu et al., 1999). P2X_7_-mediated responses in particular are biphasic (Yan et al., 2010) and characterized by sustained [Ca^2+^]_i_ elevation (Nobile et al., 2003). It is becoming increasingly clear, however, that P2 receptors cannot be studied and manipulated as individual components, but rather must be regarded as building blocks of a far more “dynamic architecture” that permits diverse functionality and flexibility (Volonte et al., 2006).

The goal of this study is to develop a universal signal-processing algorithm for MATLAB (MathWorks, Natick, MA) that would facilitate and standardize the parameter characterization of time series calcium imaging recordings containing prominent single transient responses (TR) and/or multi-peaked responses (MPR). All signals, no matter their complexity, can be reduced to a set of defined characteristics that describe the magnitude and kinetics of a given response. Based on our expertise and literature review (Tables 1, 2), we have selected the following parameters: time of onset after stimulus application (t_onset_), activation time (time for signal to increases from 10 to 90% of peak; t_10–90%_), and amplitude of response. Additionally, TRs are specifically described by their area under the curve (AUC), response duration (time between 1/2 amplitude on ascent and descent of the transient; FWHM), and decay constant of the exponential decay region of the deactivation phase of the response (τ_decay_, Figure 1B) while MPRs are described by their number of peaks (N_osc_), mean peak magnitude (E), mean periodicity (T), standard deviation of periodicity (σ_T_), oscillatory persistence (time between first and last discernable peak; *l*_osc_), and duty cycle (fraction of period during which system is active; ξ^peak^/T, where ξ^peak^ is the width of the oscillatory peaks, Figure 1C). Since MPRs can be either stochastic or deterministic (Skupin et al., 2008; Dupont and Combettes, 2009; Dupont et al., 2011), the algorithm reports two sets of MPR parameters. The first describes MPR parameters for all the peaks present, while the second set reports the MPR parameters describing the subset of coherent oscillations, to omit the influence of stochastic processes, and to focus on the deterministic properties of the signal.

Live cell recordings will inevitably contain signal contaminations arising from experimental conditions and instrumentation, including (a) photochemical effects induced by the measurement process and (b) unrelated biological processes. While these imperfections are inherent to the experimental process, dynamic processes of interest can still be extracted from these recordings. This process in itself can be complicated and highly subjective depending on the extent to which the raw data are corrupted by noise and drift. Therefore, to reliably evaluate the magnitude and kinetics of these signals, we have developed a systematic way of first identifying unwanted signal contaminations, and then removing their effects when determining the parameters of interest. Following algorithm validation, we have also investigated the effect of acidosis on ATP-mediated [Ca^2+^]_i_ responses in bone-marrow derived osteoclast precursors to demonstrate the efficacy of this algorithm in characterizing real-time cellular dynamics.

To implement the algorithm in MATLAB, the user is required to store the set of discrete sample points of the measured signal *F*(*t*), {*F*_i_}, *i* = 1, 2, ⋯ , *N*, along with the corresponding discrete time points {*t*_i_} in an Excel file (filename.xlsx). The algorithm can then be run using the MATLAB command

≫characterizeDocument(“filename.xlsx”)

The MATLAB code required to execute this command along with examples of data and scripts are provided in Supplmentary Materials (Data Sheet 1).

## Materials and methods

### Cell culture

All procedures were approved by McGill University's Animal Care Committee and complied with the ethical guidelines of the Canadian Council on Animal Care. Bone marrow precursor cells were isolated from the femur and tibia of 6 week old FVB mice (Charles River), plated at a density of 7.5 × 10^3^ on 48-well glass-bottom plates (No. 1.5 Coverslip, 6 mm glass diameter, uncoated, MatTek Corp.) and cultured for 3 days in αMEM (12,000-022, GIBCO) supplemented with 10% FBS (080152, Wisent), 1% sodium pyruvate (600-110-UL, Wisent), 1% penicillin streptomycin (450-201-EL, Wisent), 50 ng/mL MCSF (300-25, Peprotech), and 50 ng/mL RANKL according to the protocol previously described (Boraschi-Diaz and Komarova, 2016).

### Intracellular calcium measurements

After 3 days of culture, osteoclast precursors were loaded with fura2-AM, a ratiometric fluorescent calcium dye (F1221, Invitrogen), incubated at room temperature for 30 min and washed twice with physiological solution (130 mM NaCl; 5 mM KCl; 1 mM MgCl_2_; 1 mM CaCl_2_; 10 mM glucose; 20 mM HEPES, pH 7.6). The final volume of 270 μL of physiological solution at pH 7.6 or pH 7.0 was added and cells were acclimatized for 10 min to reduce the effects of mechanical agitation that resulted from fura2-AM loading and washing. 10X ATP (Sigma) solutions were prepared in physiological solution at corresponding pH and 30 μL was added after 10 s of baseline [Ca^2+^]_i_ recording to obtain a 1X dilution (i.e., final concentrations ranging from 1 μM to 10 mM ATP). [Ca^2+^]_i_ was imaged for an additional 110 s at a sampling rate of 2 images per second using a fluorescent inverted microscope (T2000, Nikon). The excitation wavelength was alternated between 340 and 380 nm using an ultra-high-speed wavelength switching illumination system (Lambda DG-4, Quorum Technologies). Regions of interest (ROI) were manually defined and the ratio of the fluorescent emission at 510 nm, following 340 and 380 nm excitation (f340/f380), was calculated and exported using the imaging software (Velocity, Improvision). All data were imported into an excel spreadsheet for subsequent analysis.

### Validation and statistical analysis

Algorithm performance was evaluated using the algorithm generated figures for 450 individual signal fitting that enabled retrospective visual examination of both response-detection and quality of parameter fitting. Manual and automated estimates were compared using a correlation plot and Bland Altman analysis (Bland and Altman, 1986) to assess the degree of correlation and agreement, respectively. For correlation analysis, the line of exact linear correlation (i.e., *y* = *x*) is plotted as a reference to assess deviation of the linear regression curve from the desired 1:1 relationship between the manual and automated estimates. For the Bland Altman analysis, we compared the automated (*a*) and manual (*m*) parameter estimates of the i^th^ recording to obtain a Z-score, given by

$$\begin{array}{l} {\text{Z}}_{\text{i}}=\frac{x_{i}-\bar{x}}{\sigma_{x}} \end{array}$$

where *x*_i_ = (*m*_i_ + *a*_i_)/2 is the average value of the estimated parameter, $\bar{x}$ is the mean value of *x* averaged over all recordings, and σ_*x*_ is the standard deviation of *x* overall recordings. Furthermore, the percent difference, Ξ_i_, of the i^th^ recording is defined by

$$\begin{array}{l} \Xi_{\text{i}}=\frac{\text{error}}{\text{average value}}\times 100\%=\frac{a_{\text{i}}-m_{\text{i}}}{x_{\text{i}}}\times 100\%. \end{array}$$

Ξ_i_ vs. *Z*_i_ were plotted to illustrate systematic biases. Negative values of Ξ_i_ were interpreted as manual estimates being greater than automated estimates, and vice versa. A quantitative estimate of the interval of agreement, within which 95% of differences lie, is defined by

$$\begin{array}{l} 95\%\text{ interval of agreement}=\bar{\Xi }\pm 1.96\sigma_{\bar{\Xi }} \end{array}$$

where $\bar{\Xi }$ is the mean percent difference over all recordings and $\sigma_{\bar{\Xi }}$ is the standard deviation of $\bar{\Xi }$.

Experimental data were expressed as means ± S.E.M. Effect of ATP treatment under control conditions was evaluated using one-way ANOVA followed with Bonferroni *post hoc* test. The effect of acidosis was evaluated using two-way ANOVA with Bonferroni *post hoc* test. Results were accepted as significant at *p* < 0.05. Statistical analysis was performed in MATLAB.

## Results and discussion

Although the notation used throughout the text implies that the recorded signals are fluorescence, the methodology remains the same for any other type of signals. For fluorescent recordings, the measured fluorescence *F* may consist of multiple parts: the drift, TR including the activation and deactivation phases, and the superimposed MPR. It can be expressed as the sum of the actual signal, *F*_*true*_, and the normally distributed noise with a standard deviation σ.

(1) $$F(t)=F_{true}(t)+\;N\left(0,\sigma \right).$$

To characterize parameters that reliably reflect *F*_*true*_(*t*), *F*(*t*) is first preprocessed to remove the effects of noise (Section Noise Characterization) and to estimate the contributions of drift to *F*_*true*_(*t*) (Section Baseline Drift). Next, the activation phase of TR is fit while simultaneously refining the estimated contribution of the drift. This approach allows us to determine if the recording is consistent with the expected model of a TR superimposed on a drifting baseline (i.e., whether activation phase is followed by a deactivation phase, Section Activation Fitting). If a TR is detected, we proceed by fitting the full set of TR model parameters simultaneously with the drift parameters [Section Transient Response (TR) Model]. In the case where there remain multiple significant deviations in the data from the TR model, we investigate and characterize the presence of oscillatory MPRs (Section Multi-Peaked Responses). The fitting of the TR is refined to remove the effects of the multiple peaks on the initial fit (Section Identifying Coherent Oscillations), in order to provide the best estimate of the baseline around which the MPRs oscillate. The deviations resulting from this secondary fitting of the TR are then analyzed to determine those resulting from coherent oscillatory processes (Section Characterizing Oscillatory Parameters). At each step throughout the fitting procedure, an updated estimate of the optimal set of parameters (e.g., the drift parameters) is obtained. These parameters are then used in a feed-forward manner, where the optimal set of parameters of the preceding fit is used as an initial guess for the subsequent step. This ensures that the algorithm produces high-fidelity fittings. Finally, the algorithm performance and utility is demonstrated with a new data set describing the effect of acidosis on ATP-induced calcium signaling in osteoclast precursors (Section Application to Pathophysiology).

### Noise characterization

The first step in the processing of data is to evaluate four values that will be used for the remainder of the text: the derivative (*û*) of the noisy signal, the standard deviation of noise (σ_−_), indices at which this noise is not prevalent (*j*), and the noise-to-signal ratio (ϕ). *û* will be used in Section Drift Delimitation to separate the TR from the underlying drift. Data points excluded by *j* (i.e., noise) will be omitted. The methodology detailed throughout the following section is an iterative procedure. In the instances where those quantities are used, we are referring to the value determined by the final iteration of the procedure.

#### Euler-lagrange formalism

The presence of noise in a recording renders naïve methods of derivative estimation inadequate (Chartrand, 2011). This is particularly exacerbated by the intermittent presence of large amplitude noise (spikes) related to the use of high gain settings on instrumentation. To reliably delimit (i.e., define the boundaries of) the drift in a recording of a noisy transient signal (Section Drift Delimitation), we have adapted the total-variational (TV) technique commonly used to estimate the first derivative of a signal contaminated with various types of noise (Chartrand and Staneva, 2008; Chartrand and Wohlberg, 2010; Chartrand, 2011). This technique performs better than the low-pass filter in distinguishing the drift from the transient response, as it does not indiscriminately remove high frequency components that affect the overall trend of the signal.

Our TV-based methodology seeks a function, $\hat{\text{u}}\left(\text{t}\right)$, which represents the derivative of F_true_(t), that solves the optimization problem.

(2) $$\begin{array}{l} \underset{u}{\min}\alpha \overset{L}{\underset{0}{\int }}|\frac{du}{dx}|dx+\frac{1}{2}\overset{L}{\underset{0}{\int }}{\left|\left(Au\left(x\right)-F\left(x\right)\right)\right|}^{2}dx \end{array}$$

The first term in Equation (2) is a regularization term which penalizes sudden changes in the derivative (to make the fitting smooth), the second term is an *L*^2^ data fidelity term, where *A* is the anti-differentiation operator (*Au* ≈ *F*_*true*_), and α is a parameter dictating the balance between the two terms. In order to solve this minimization problem, we have to find the stationary solution to the following equation

(3) $$\begin{array}{l} u_{t}\left(x\right)=\alpha \frac{d}{dx}\frac{u^{\prime }\left(x\right)}{\left|u^{\prime }\left(x\right)\right|}-A^{T}\left(Au\left(x\right)-F\right) \end{array}$$

derived from the Euler-Lagrange equation associated with Equation (2), where $A^{T}v\left(x\right)=\overset{L}{\underset{x}{\int }}vdx$ is the *L*^2^ -adjoint of *A*. Within the context of ratiometric fluorescent dyes (such as Fura2 AM used for [Ca^2+^]_i_ recordings in this study) the recorded signal is the ratio of two Poisson random variables. The variance and the mean of such a signal follow a complex, and seemingly non-linear, function of the photon count rates at each wavelength. Since these rates are assumed to be unknown *a posteriori* in a recording, we cannot accurately determine how the noise is distributed. However, we will assume that instrumentation and experimental settings contribute to a noise distribution that is approximately Gaussian. Moreover, in the specific case of ratiometric dyes (Section Intracellular Calcium Measurements), we have found that true noise distribution is a complex function of time, but can be represented reliably using a time-dependent Gaussian noise model. Therefore, to reduce data-fidelity and conversely increase regularity in the regions of highest amplitude noise while accurately reproducing data in regions of lowest noise, we find instead a stationary solution to the following equation

(4) $$\begin{array}{l} u_{t}\left(x\right)=\alpha \frac{d}{dx}\frac{u^{\prime }\left(x\right)}{\left|u^{\prime }\left(x\right)+\varepsilon \right|}-\frac{A^{T}\left(Au\left(x\right)-F\right)}{\psi \left(x,u\right)+\eta }, \end{array}$$

where ψ(x, u) is an iteratively determined weighting function (as described below), and ε and η, are parameters introduced to avoid dividing by zero.

At the *n*^th^ iteration of the algorithm, we solve for *u*^*n*+1^ by setting the left-hand-side of Equation (4) to zero, and linearizing the problem through substituting every *u* appearing in the denominators by the value of *u*^*n*^ obtained from the previous iterate. For a more detailed descritption of the means used to solve this type of problem (see Vogel, 2002; Chartrand and Staneva, 2008; Chartrand and Wohlberg, 2010). It is known that an appropriate choice of the denominator offsets, ε and η, is necessary to produce acceptable minimizations (Chartrand, 2007), yet this choice is rarely considered beyond their status as parameters that must be tweaked to obtain acceptable results (Li et al., 2007; Chambolle et al., 2009; Oh et al., 2013). In what follows, we detail a methodology on how to determine the parameters α, ε, η, and the function ψ(*t*), based on the data *F*(*t*), and the derivative estimate *u*(*t*).

#### Dynamic determination of total-variational parameters

Given a set of fluorescence recordings {*F*_i_} of length *N*, at the *n*^th^ iterate of the regularization algorithm, we identify the set of indices $j=\left\{\text{i}=1,2,\cdots \;,\text{N}|\psi^{\text{n}}\left(t_{\text{i}}\right)\neq \infty \right\}$ (as explained in Section Removal of Noise Spikes) whose data are not likely dominated by noise and thus should contribute to the fidelity term of Equation (4). Letting Δ_i_ = *F*_i_ − *F*_i − 1_, we can define the weighting sequence $g_{\text{i}}=1-\left|\Delta_{\text{i}}\right|/\underset{\text{i}}{\text{max}}\;\left|\Delta_{\text{i}}\right|$ in order to provide an upper bound on the noise of the signal, given by

$$\begin{array}{l} \sigma_{+}^{n}=\frac{\underset{j}{\sum }|\Delta_{j}|g_{j}}{\underset{j}{\sum }g_{j}}. \end{array}$$

The weights *g*_i_ will tend to zero as Δ_i_ approach their maximum, and converge to a positive number (< 1) as |Δ_i_| approach their minimum. We can thus conclude that the weighted average of |Δ_*j*_| will identify the smallest differences as being the most informative of the magnitude of noise. Large discrete differenecs, whether they result from transient increases in the noise level or from the fact that the signal is non-stationary, contribute only modestly to the estimate $\sigma_{+}^{n}$. On the other hand, we can also estimate a lower bound on the noise using

(5) $$\begin{array}{l} \sigma_{-}^{n}=mean\left(\left|\zeta_{j}^{n}\right|\right), \end{array}$$

where $\zeta_{\text{i}}^{n}=F_{\text{i}}-{\left(Au^{n}\right)}_{\text{i}}$, i.e., by taking the difference between the data and the cumulative integral of *u*. Because we use the discrete differences Δ_i_ as our initial solution: $u_{\text{i}}^{0}=\Delta_{\text{i}}$, the integral of *u*^*n*^ (*n* > 0) will likely diverge away from the data with each succesive iteration of the algortithm due to the action of the regularization term in Equation (4). This tends to result in $\sigma_{-}^{n}$ being smaller than $\sigma_{+}^{n}$, although this is not always strictly true. The use of the two different estimators for the noise allows for a more robust performance of the methodology, as both estimators are prone to becoming inaccurate in different scenarios. With an estimate of the noise, we can also estimate the noise-to-signal ratio by

$$\begin{array}{l} \phi^{n}=\frac{\sigma_{+}^{n}}{\max\left(Au^{n}\right)}. \end{array}$$

The value of ϕ is a critical parameter in our algorithm as it discreminates between small and large values of various quantities. For example, it is employed to calculate an appropriate value of ε, defined in Equation (4), based on the scale of variations of small values of *u*′, as follows

$$\begin{array}{l} \varepsilon^{n}=std\left(\left\{|{u^{\prime }}_{r}|:|{u^{\prime }}_{r}|\leq {\left(\phi^{n}\right)}^{2}\underset{\text{i}}{\max}\left(\left|{u^{\prime }}_{\text{i}}\right|\right),r\in i\right\}\right). \end{array}$$

The calculation of η requires defining another weighting sequence $h_{i}=|{u^{\prime }}_{i}|/\underset{\text{i}}{\text{max}}|{u^{\prime }}_{i}|$, which tends to zero when *u* is the most regular, as well as an estimate for the upper bound on the total error, χ, between the integral of *u* and the data in the least regular regions of the solution, given by

$$\begin{array}{l} \chi^{n}=\frac{\underset{\text{i}}{\sum }h_{\text{i}}|\zeta_{\text{i}}^{n}|}{\sigma_{-}^{n}}. \end{array}$$

When χ is small, η must be made large enough to improve the smoothness of the fitting (at the expense of data-fidelity). This can be acheived by making η a decreasing function of χ. However, if a recording does not contain any rapid jumps or noise spikes (but is nonetheless noisy), such a relation between χ and η will not be sufficient to infer a proper choice of η given χ. Thus, we must include another term independent of χ which will produce modest data-fidelity for signals dominated by drift. We therefore define η to be

$$\begin{array}{l} \eta^{n}=\frac{\exp}{\sigma_{+}^{n}}\left[\begin{array}{l} -(\frac{\chi^{n}+1}{2\sigma_{-}^{n}}\text{max}(\{\left|F_{i}-F_{\text{i }-\;1}\right|-std(\{\left|F_{r}-F_{r\;-\;1}\right|\\ \;{:r=2,\ldots ,N\}):i=2,\ldots ,N\}))}^{\frac{\sqrt{\chi^{n}}}{2}} \end{array}\right]\\ \;+\;mean\left(\underset{d\subset \left\{\delta^{n}\right\}}{arg\;max}\{|d{|}_{\ell^{1}}:\text{mean}(d)\geq 3\text{std}(d)\}\right). \end{array}$$

which includes the smallest non-zero scale of the weighted differences $\delta_{\text{i}}^{n}=\left(1-h_{\text{i}}^{n}\right)\Delta_{\text{i}}^{n}$.

TV methods tend to smooth the fit when there are large amplitude jumps in the data, or where *u* is large. If a single large jump dominates the derivative, this can lead to excessive local smoothing, which can be resolved by having enhanced data-fidelity at that point. On the other hand, in the presence of large jumps in the data, small noise-driven fluctuations may be under-regularized. In this case, data-fidelity at these points must be reduced. The function ψ allows for local enhancement or reduction of data-fidelity. Unlike ε, η does not depend on ϕ, but data-fidelity must through the weighting function ψ (by making ψ proportional to ϕ). Therefore, we define ψ to be

(6) $$\begin{array}{ll} \psi_{\text{i}}^{n}={\left(\phi^{\sqrt{\chi^{n}+2}}\right)}^{J_{1}}+\phi^{J_{2}}, & \; \end{array}$$

where

$$\begin{array}{l} J_{1}\;=\;\left[\frac{1}{{\left(\sigma_{+}^{n}\right)}^{1+\sqrt{\phi^{n}}}}\sqrt{\frac{\left|u_{\text{i }+\;1}^{n\;-\;1}\right|}{\min\left(\left\{\left|u_{r}^{n\;-\;1}\right|:u_{r}^{n-1}\neq 0\right\}\right)}}\;\right],\\ J_{2}\;=\;\left[\sqrt{\frac{\left(\frac{1}{\tau_{\max}}+\frac{1}{3}\right)\sigma_{+}^{n}}{\left(\chi^{n}+1\right)\left(\omega_{\text{i}}+\delta_{\text{i}}\right)}}+\frac{mean\left(\left|u^{n\;-\;1}\right|\right)}{\left|\max\left(\left|\upsilon^{n}\right|\right)-\left|\upsilon_{\text{i}}^{n}\right|+\phi^{n}\right|}\right], \end{array}$$

$\tau_{\max}^{n}=\left(mean\left(\left|\zeta_{j}^{n}\right|\right)+std\left(\left|\zeta_{j}^{n}\right|\right)\right)/\sigma_{+}^{n}$ is a iterative error scale parameter, $\upsilon_{\text{i}}^{\text{n}}={\text{u}}_{\text{i}+1}^{\text{n}-1}+{\left(1-{\text{h}}_{\text{i}+1}\right)}^{2}{\text{u}}_{\text{i}-1}^{\text{n}-1}$ is an estimator of *u*_i_ based on the adjacent values of *u*, and ω_i_ is a three-point moving average of δ_i_. The exponent *J*_1_ emphasizes data-fidelity (regularity) when the derivative of the following point is large (small), whereas the exponent *J*_2_ emphasizes data-fidelity when Δ_i_ are small or when the derivative is near its maximum. When Δ_i_ is large and *u*_i−1_, *u*_i_ are regular, on the other hand, *J*_2_ is small allowing regularity to propagate forward into regions of signal possessing large ampltidue noise (i.e., where the data is not informative). The balance between the two effects of *J*_2_ along with the χ -dependent terms of Equation (6) produce an acceptable compromise between the regularity of the fit and data-fidelity for recordings across a wide range of signal-to-noise ratio and extremely varied dynamics. Finally, once the maximum relative change between two iterations of the procedure becomes less than $\sqrt{\phi }$, we consider the solution to have achieved quasi-stationarity and terminate the procedure.

#### Removal of noise spikes

**Figure 2:**
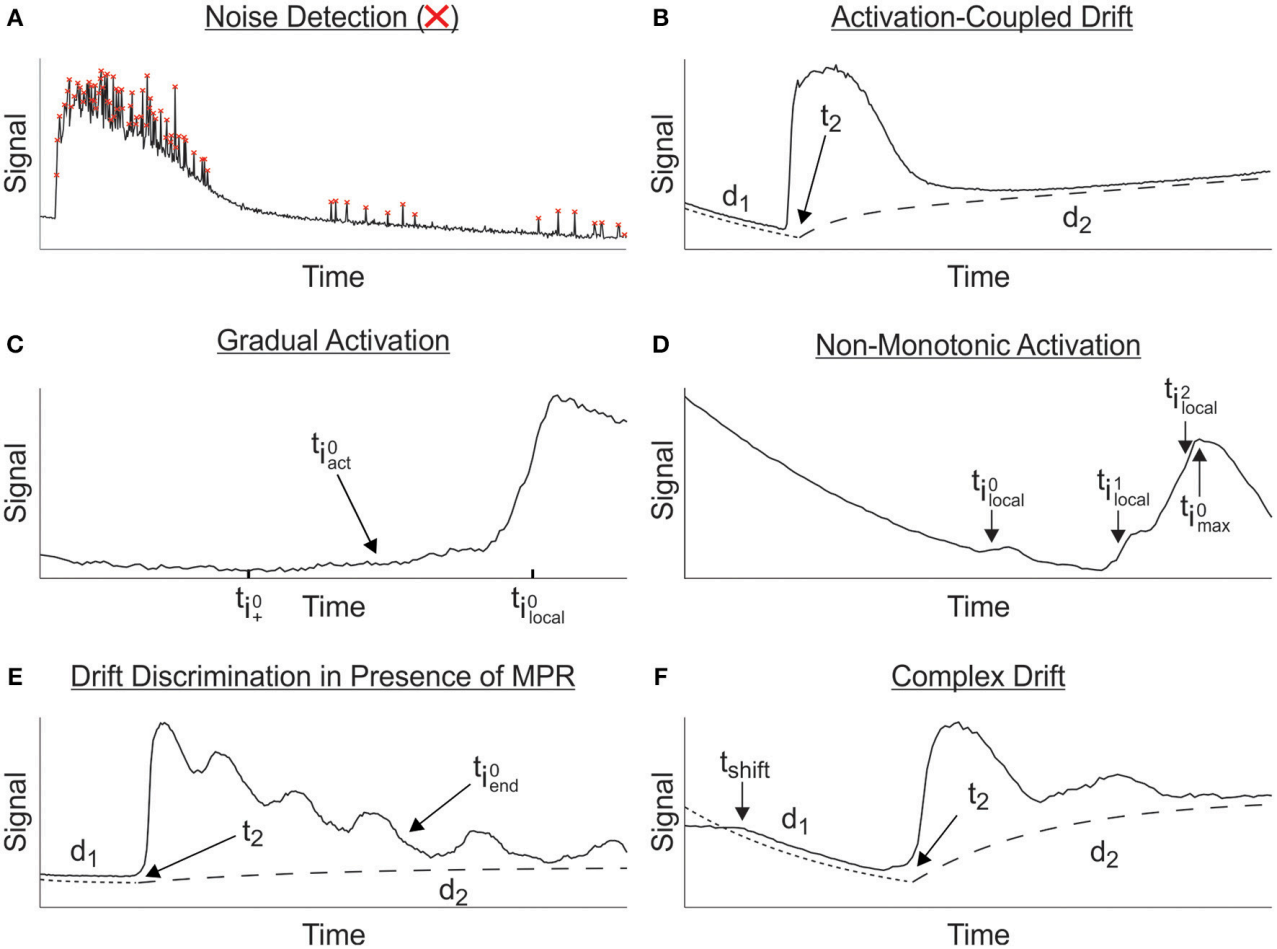
**Multitude of features that must be considered during parameter characterization. (A)** Experimental recordings are often contaminated with noise; red crosses represent noise detected and corrected. **(B)** Activation-coupled drift; Trajectory of baseline drift, d_1_, can shift to secondary drift, d_2_ at time t_2_. **(C)** Determination of activation in the presence of gradual activation. Region of activation is restricted to $\left[t_{{\text{i}}_{+}^{0}},t_{{\text{i}}_{local}^{0}}\right]$, within which the most likely point of activation, $t_{{\text{i}}_{act}^{0}}$, is statistically resolved. **(D)** Non-monotonic activation. Spurts of negligible activity, represented by the local maxima at $t_{{\text{i}}_{local}^{0}}$ and $t_{{\text{i}}_{local}^{1}}$, are disregarded as points of activation, in favor of $t_{{\text{i}}_{local}^{2}}$ which is characterized by the max peak, $t_{{\text{i}}_{\text{max}}^{0}}$. **(E)** Drift discrimination in cases where MPRs are superimposed on TRs. Underlying TR serves as a non-stationary baseline around which the MPR will oscillate within $\left[t_{2},t_{{\text{i}}_{end}^{0}}\right]$ until its contribution becomes negligible and the baseline drift, d_2_, dominates the MPR baseline. **(F)** Complex drift; Change in the baseline drift, t_*shift*_, prior to onset of TR is ignored in favor of d_1_ after the reweighting procedure outlined in Section Drift Fitting.

When a recording exhibits intermittent periods of high amplitude noise (noise spikes), the data contaminated by these noise spikes is minimally informative. Detecting them allows for determining the indices *j* (Figure 2A). Within our regularization algorithm, this is done by (a) setting ψ to infinity at those time points in such a way that Equation (4) only penalizes irregularity at these time points, and (b) determining the fit at these points based on the surrounding (reliable) data. After each iteration, *n*, of the regularization algorithm, a smoother fit, *Au*^*n*^, of the data is obtained. We also obtain a criterion that determines whether or not each point represents a noise spike based on a comparison between the residual differences, ζ^*n*^, and a chosen threshold value. This threshold is specified using the parameter $\tau_{rm}^{n}$, given by

$$\begin{array}{l} \tau_{rm}^{n}=\left(1-\xi \right)+\xi \tau_{\max}^{n}, \end{array}$$

where $\xi =\exp\left(-\left(\left(\sigma_{+}^{n}-\sigma_{-}^{n}\right)/\max\left\{\sigma_{+}^{n},\sigma_{-}^{n}\right\}\right)-{\left(\chi^{n}\right)}^{2}/N\right)$ is a convergence parameter for the noise rejection method. Positive $\zeta_{\text{i}}^{n}$ are rejected if they are greater than $\tau_{rm}^{n}\sigma_{+}^{n}$, while negative $\zeta_{\text{i}}^{n}$ are rejected if they are less than ${\left(\tau_{rm}^{n}\right)}^{2}\sigma_{+}^{n}$. The use of two thresholds is due to the asymmetry of the Poisson statistics underlying data collection using photodetectors. Rejection is achieved by setting ψ_i_ = ∞, which serves as the basis for defining the set of indices *j* (i.e., $j=\left\{\text{i}=1,2,\cdots \;,\text{N}|\psi^{\text{n}}\left(t_{\text{i}}\right)\neq \infty \right\}$ as stated before).

### Baseline drift

When fitting data to specific functional forms, it is important to take into account temporal drifting of the baseline in a signal. The specific nature of the processes underlying this drift are not of particular interest here. Rather, we consider them as nuisance trends and aim to remove their effects from the data.

#### Drift model

Some drifts are quite slow compared to the timeframe of the experimental recording, and thus can be fairly well represented by linear functions, whereas others are fast and better represented by an exponential decay function. Since, in a given recording, a number of processes will result in the observed drift, we postulate that, the drift throughout the signal can be well fit to a combination of linear and exponential functions

(7) dι(t;aι,τι,mι)=aι[1−exp(−tτι)]+mιt

where, *a*_ι_, τ_ι_, and *m*_ι_ are the exponential amplitude, exponential time constant and the slope of the linear component of the ι^th^ drift within the signal, respectively (examples of fitted drifts shown in Figures 2B,E,F). With an appropriate choice of data, these are determined using the least squares fitting as discussed in Section Drift Fitting. We have found numerous cases where either the linear or exponential components were not justifed. However, this knowledge is unavailable to us prior to conducting manual or automated analysis of the data, and we cannot assume *a priori* a less general form than Equation (7). Thus, we have to rely on the fitting to optimize the contribution of the two compoenets in a data-dependent manner.

It is possible for the signal to exhibit (multiple) drifts with different trends separated perhaps by a TR. In order to capture this effect in a signal, we use a global drift model that combines the intial and secondary drifts (Figure 2B) in a semi-piecewise manner in which the initial drift, d_1_, continues to contribute to the overall observed drift and succesfully captures the global behavior. This can be written as

(8) $$D\left(t\right)=\{\begin{array}{cc} d_{1}\left(t\right)+z & if\;t<t_{2}\\ d_{1}\left(t\right)+d_{2}\left(t-t_{2}\right)+z\; & \;if\;t\geq t_{2} \end{array},$$

where *t*_2_ is the time at which the secondary drift, d_2_, begins and *z* is the offset at *t* = 0. Rather than assuming that the drift is similar for all recordings and attempting to construct a standard curve, we assume that a few points in each recording are highly informative of the drift in the baseline.

#### Drift delimitation

To fit the drift model to the corresponding portion of the recorded signal, it is necessary to delimit the boundaries of TR by identifying the start of activation and end of deactivation. Firstly, we will aim to estimate the point in time at which the TR of a recording begins. Experimental TRs rarely activate abruptly and simultaneously for a field of imaged cells. Many factors play a role in the heterogeneity of responses observed such as variable diffusion fronts of applied agonist or heterogenous receptor expression among cells. These effects can manifest as very gradual rises or additional small amplitudes prior to a certain activation threshold being surpassed and a rapid activation phase being observed (Figures 2C,D). Therefore, analyzing the activation times of all components of the biological unit manually can be subjective. While a simple threshold value in the signal can be effective in detecting activation, the choice of the threshold requires some knowledge of the amplitude of the noise σ and is complicated by the presence of drift in the signal. Instead, we determine the end points of the time intervals dominated by the drift through statistical analysis of an estimate of the first derivative of the signal *û*(*t*). In other words, to distinguish the drift from TR, the derivative of the latter must change in a way that is more statisically significant than that of the former. The methodology used to obtain the estimate for the derivative is detailed in Section Noise Characterization.

Using the estimated time derivative, we aim to determine (i) the earliest possible time of activation $t_{{\text{i}}_{\text{act}}^{0}}$ (defined as the last time point exhibitng a significant increase in the first derivative before it reaches its maximum value), (ii) the time at which the activation reaches its maximum value *t*_i_*max*__, and (iii) the time at which the deactivation ends and the signal is once again dominated by the drift $t_{{\text{i}}_{\text{end}}^{0}}$.

- Assuming that the estimated first derivative during activation reaches a local maximum, we can find the most significant local maxima of *û* at the location
  $$\begin{array}{l} {\text{i}}_{local}=\left\{i:{\hat{u}}_{\text{i}}>std\left(\hat{u}\right),{\hat{u}}_{\text{i}}\geq {\hat{u}}_{\text{i }-\;1}\text{and}{\hat{u}}_{\text{i}}\geq {\hat{u}}_{\text{i }+\;1}\right\}, \end{array}$$
- where we denote the first significant local maximum by $i_{local}^{0}\;=\;\min\left\{{\text{i}}_{local}\right\}$. By focusing on the portion of the signal containing the first drift and the activation phase of the TR, we restrict our attention to the time interval in which the first derivative *û*(*t*) is non-negative to disentangle the effects of activation and drift in the data. In other words, we restrict our analysis to $\left[t_{{\text{i}}_{+}^{0}},t_{{\text{i}}_{\text{local}}^{0}}\right]$, where the derivative is non-negative and
  $$i_{+}^{0}=\underset{\text{i}\in 0,\ldots ,{\text{i}}_{\text{local}}^{0}}{\text{min}}\left\{i:{\hat{u}}_{r}\geq 0\;\forall r=i,\ldots ,i_{local}^{0}\right\}.$$
- Without prior knowledge about the sign of the derivative of the baseline drift, we cannot conclude that $i_{+}^{0}$ corresponds to the beginning of activation. To resolve this issue, we employ a statistical test to determine the likely time at which the activation occurs, located at the index
  $$i_{act}^{0}=\underset{\text{i}\in {\text{i}}_{+}^{0},\ldots ,{\text{i}}_{\text{local}}^{0}}{\text{max}}\left\{i:{\hat{u}}_{\text{i}}<std\left(\left\{{\hat{u}}_{{\text{i}}_{+}^{0}},\ldots ,{\hat{u}}_{{\text{i}}_{\text{local}}^{0}}\right\}\right)\right\}.$$
- This methodology, based on the properties of the derivative around its first significant local maximum, generally picks out the first visually unambiguous activation (Figure 2C). As a result, it may be necessary to trim recordings where there are (large amplitude) artifacts prior to the activation of interest.
- TRs may be produced by the action of multiple active units (e.g., different receptor species) within the biological system under consideration, each having distinct properties and activation times. This leads to multiple delayed activations taking place over a broad range of time (Figure 2D). Due to the superposition of the drift in the baseline with these responses, it is entirely possible for recordings to be contaminated by strongly decreasing drift and for the expected maximum TR to not coincide with the actual maximum of the data. Thus, we have developed a method to search for the visually most likely point at which the TR reaches its maximum in the presence of a drift. For each one of the *N*_*local*_ significant local maxima of the first derivative along the activation phase, we find the location of the previous local minimum of the TV estimate of the data using
  $$\begin{array}{ll} i_{local}^{min}=\left\{\underset{n\;<\;r}{\max},\left\{n:\hat{u}\leq 0\right\}:r\in {\text{i}}_{local}\right\} & \; \end{array}$$
- as well as the location of the next local maximum at
  $$\begin{array}{ll} i_{local}^{max}=\left\{\min_{n\;>\;r}\left\{n:\hat{u}\leq 0\right\}:r\in {\text{i}}_{local}\right\}. & \; \end{array}$$
- From the positions of the local extrema of the data, we can estimate the value of the baseline drift from each local minimum to the next local maximum using the linear extrapolation
  (9) $$\begin{array}{r} \nu_{-}=\left\{\begin{array}{c} {\left(A\hat{u}\right)}_{q}+\left(t_{r}-t_{q}\right)\frac{{\hat{u}}_{r\;-\;1}\;+\;{\hat{u}}_{r}}{2}\;:q={\left(i_{local}^{min}\right)}_{s},\\ \;r={\left(i_{local}^{max}\right)}_{s},s\in \left\{1,\ldots ,N_{local}\right\} \end{array}\right\} \end{array}$$
- where *A* is the operator of antidifferentiation with *Au* ≈ *F*_*true*_ (see Equation 1). Based on this, we then estimate the average rate of activation for each significant local maximum of the derivative according to
  $$\begin{array}{l} \mu =\left\{\begin{array}{c} \frac{{\left(A\hat{u}\right)}_{r}-{\left(\nu_{-}\right)}_{s}}{t_{r}-t_{q}}\;:q={\left(i_{local}^{min}\right)}_{s},r={\left(i_{local}^{max}\right)}_{s},\\ \;s\in \left\{1,\ldots ,N_{local}\right\} \end{array}\right\} \end{array}$$
- and select the first local maximum at the location
  $$\begin{array}{l} i_{max}^{0}=min\left\{\begin{array}{c} r:\mu_{r}\geq mean\left(\mu \right)-3\cdot std\left(\mu \right)\text{and }\nu_{r}\\ \;>mean\left(\nu \right)-std\left(\nu \right) \end{array}\right\}. \end{array}$$
- which has an average rate and magnitude within a statistically acceptable range (that excludes small outliers). $t_{{\text{i}}_{\text{max}}^{0}}$ is the time point at which the derivative reaches a local maximum. It may differ from the one that corresponds to the local maximum immediately following the first activation time point $t_{{\text{i}}_{\text{act}}^{0}}$ (Figure 2D). This is because $i_{act}^{0}$ depends solely on the derivative around its first local maximum, while $i_{max}^{0}$ takes into account an approximation to the average rate of change around all local maxima; the local maximum after $i_{act}^{0}$ should only differ from $i_{max}^{0}$ when there is a succession of activations and the first does not have the largest rate of activation.
  Starting from this local maximum of the derivative, we seek the location of the first point in time $t_{{\text{i}}_{\text{max}}^{\text{end}}}$ where the change in the signal drops below the estimated noise level σ_−_ (see Section Dynamic Determination of Total-Variational Parameters)
  (10) $$i_{max}^{end}=\underset{\text{i}\in {\text{i}}_{\text{max}}^{0},\ldots ,\text{N}}{min}\left\{i:{\hat{u}}_{\text{i}}\cdot \left(t_{\text{i}}-t_{\text{i }-\;1}\right)<\sigma_{-}\right\},$$
- and thus isolate the time interval in which the most significant activation occurs. Having isolated the most significant activation, we finally arrive at the location of the first estimate of the time where the response reaches its maximum
  $${\text{i}}_{max}=min\left\{\underset{\text{i}\in {\text{i}}_{\text{max}}^{0},\ldots ,{\text{i}}_{\text{max}}^{\text{end}}}{\arg\max}{\left(A\hat{u}\right)}_{\;i}\right\}.$$
- Having identified the time at which activation is likely to begin $t_{{\text{i}}_{\text{act}}^{0}}$, we can now assume that the signal prior to this point is the drift. If the recording is of a sufficiently long duration, the response will return to baseline and the end of the recording should once again be dominated by the (secondary) drift (Figure 2A). To account for this drift, we need to estimate the time duration of deactivation. This is done in a manner nearly identical to how we determined the first time of activation $t_{{\text{i}}_{\text{act}}^{0}}$. However, due to the possibility of having MPR after the initiation of TR (Figure 2E), we cannot restrict ourselves to time intervals in which the first derivative is non-positive. To solve this issue, we define a set of time points after the presumed maximum of TR, located at i_*decay*_ = {i_*max*_, …, *N*}, and construct a measure
  $$\begin{array}{l} n_{end}=\underset{n\in \left\{3,2,1,0\right\}}{max}\left\{\begin{array}{c} n:\exists |{\hat{u}}_{\text{i}}|-mean\left(\left|{\hat{u}}_{{\text{i}}_{decay}}\right|\right)\\ \;>n\cdot std\left(\left|{\hat{u}}_{{\text{i}}_{decay}}\right|\right),i\in {\text{i}}_{decay} \end{array}\right\} \end{array}$$

to quantify how far the derivative deviates from its mean during the decay. This measure allows us to robustly detect the location of the time point where TR is negligible

$$\begin{array}{l} i_{end}^{0}=\underset{\text{i}\in {\text{i}}_{\text{decay}}}{min}\left\{i:|{\hat{u}}_{\text{i}}|-mean\left(\left|{\hat{u}}_{{\text{i}}_{decay}}\right|\right)>n_{end}\cdot std\left(\left|{\hat{u}}_{{\text{i}}_{decay}}\right|\right)\right\}. \end{array}$$

The time point $t_{{\text{i}}_{\text{end}}^{0}}$ is then used to determine the start of the new drift.

#### Drift fitting

To isolate the TR from the drift, it is necessary to generate an accurate fit for the drift. This is achieved by employing a succession of least square fits that progressively incorporates more data and models that account for additional components of the signal (including activation and deactivation phases of TR). The first step in this successive least-square-fitting method is to obtain preliminary estimates of the parameters θ_*drift*_ = [*a*_1_, τ_1_, *m*_1_, *a*_2_, τ_2_, *m*_2_] of the drift model. The MATLAB implementation of the non-linear least squares method (Marquardt, 1963; Moré, 1978) is used. More specifically, we initially minimize the error function between the drift model and the data

(11) $$\begin{array}{l} S_{drift}^{0}\left(\theta_{drift}\right)=\underset{k}{\sum }{\left(D\left(t_{k};\theta_{drift}\right)-F_{k}\right)}^{2}, \end{array}$$

where the set of indices *k* is defined by $k=\left\{\left\{1,\ldots ,i_{act}^{0}\right\}⋃\left\{i_{end}^{0},\ldots ,N\right\}\right\}⋂j$ ($i_{act}^{0}$ and $i_{end}^{0}$ are as defined in Section Drift Delimitation while *j* is defined in Section Removal of Noise Spikes). We denote the first estimate of parameters obtained from the minimizaiton of Equation (11) by $\theta_{drift}^{0}$. There is no guarantee, however, that the drift model described by Equation (8) can accurately represent the actual drift in the baseline. The emergence of drifting trends which are not related to the onset of TR (Figure 2F) may require the inclusion of more than two functions of the type described in Equation (7), yet the decision to include more drift terms, or alternatively to truncate the signal, would require manual intervention. To circumvent this limitation, we perform a second fit where the individual terms in Equation (11) are weighed according to a weight function *w*, and the first derivative of the data is taken into account. Although the set of parameters $\theta_{drift}^{0}$ will be able to produce the “general” trends in the data before and after TR, the presence of multiple drifting trends necessitates the use of the derivative estimate, *û*, to identify the segment(s) of the signal that actually follow the trend described in Equation (8) and remove the effects of others. The inclusion of *û*, however, in the sum of squared errors can lead to erroneous fittings when the drift is not well represented by Equation (8). The weight function *w* alleviates this problem by preventing the linear and exponential trends in Equation (7) from growing unjustifiably large.

To determine *w*, we require that it approaches zero when the match between *û* and $Ḋ\left(t;\theta_{drift}^{0}\right)$ is minimal. To achieve this, we choose *w* to depend on $y=|\hat{u}-Ḋ\left(t;\theta_{drift}^{0}\right)|$ as follows

$$\begin{array}{l} w_{k}=exp\left(\frac{-y_{k}^{2}}{mean\left(y_{k}^{2}\right)}\right). \end{array}$$

We then apply the non-linear least squares fitting procedure to minimize the error function

$$\begin{array}{l} S_{drift}(\theta_{drift})=\sum_{k}w_{k}\left[\begin{array}{l} {\left(D\left(t_{k};\theta_{drift}\right)-F_{k}\right)}^{2}\\ \;\;\;\;+\;{\left({\hat{u}}_{k}-Ḋ\left(t_{k};\theta_{drift}\right)\right)}^{2} \end{array}\right], \end{array}$$

and use $\theta_{drift}^{0}$ as an initial condition for the fitting procedure.

### Activation fitting

For the activation phase of the response, we use the model

$$\begin{array}{lll} g_{act}\left(t;A_{act},\beta ,n_{act},m_{act}\right) & = & A_{act}\frac{t^{n_{act}}}{t^{n_{act}}+\beta^{n_{act}}}\\ \;+\;m_{act}\overset{t}{\underset{0}{\int }}\frac{x^{n_{act}}}{x^{n_{act}}+\beta^{n_{act}}}dx. \end{array}$$

where *A*_*act*_ is the maximum of the Hill function, *n*_*act*_ is the Hill coefficient, β is the time at which the the Hill function reaches its half maximum, and *m*_*act*_ is the slope of the quasi-linear function that accounts for the trend which dominates at the end of activation. The Hill function allows for a rapid rise and switch in convexity due to many biological units being activated at once, whereas the linear trend allows for a delayed and slower rise induced by more units being progressively recruited into the generation of the signal. The values of *A*_*act*_ and *m*_*act*_ determine the magnitude of these two trends, whereas *n*_*act*_ and β affect primarily the timescales of switching between the two. The time at which the activation phase begins is denoted as *t*_*on*_, and its estimate is confined to the time interval $\left[t_{{\text{i}}_{\text{act}}^{0}},t_{{\text{i}}_{\max}}\right]$. In order to obtain a preliminary estimate of the values of these parameters and those of the drift function, θ_*act*_ = [*t*_*on*_, *A*_*act*_, β, *n*_*act*_, *m*_*act*_, θ_*drift*_], we minimize the sum of square errors between the activation data and the model along with their derivatives, given by the function

(12) $$\begin{array}{r} S_{act}\left(\theta_{act}\right)=\underset{j}{\sum }\left[\begin{array}{c} \phi^{2}{\left(F_{j}-G_{act}\left(t_{j};\theta_{act}\right)\right)}^{2}\\ +\;\phi {\left({\hat{u}}_{j}-{Ġ}_{act}\left(t_{j};\theta_{act}\right)\right)}^{2} \end{array}\right]+S_{drift}\left(\theta_{drift}\right) \end{array}$$

where ϕ is the noise-to-signal ratio as defined in Section Dynamic Determination of Total-Variational Parameters, and

$$\begin{array}{l} G_{act}\left(t;\theta_{act}\right)\\ =\{\begin{array}{cc} D\left(t;\theta_{act}\right)+g_{act}\left(t-t_{on};\theta_{act}\right) & if\;t_{on}\leq t<t_{max}\\ D\left(t;\theta_{act}\right) & otherwise \end{array} \end{array}$$

is our activation data model that takes into account the effect of the drift *D* (defined in Equation 8) on the perceived activation. To abrogate the influence of noise spikes on measured parameters, the sum of squares in Equation (12) is evaluated at the set of indices *j* (defined in Section Dynamic Determination of Total-Variational Parameters) which are not dominated by noise. Moreover, we use ${\hat{\theta }}_{drift}^{act}$ to denote the set of drift parameters obtained from the minimization of Equation (12).

#### Signal detection

In order for the algorithm to resolve whether there is a discernable TR present in *F*_*true*_(*t*), three conditions must be satisfied: (i) i_*local*_ must be a non-empty set, (ii) the initial drift estimate

$$\begin{array}{l} D\left(t;\underset{\theta \in \left\{{\hat{\theta }}_{drift},{\hat{\theta }}_{drift}^{act}\right\}}{\mathrm{argmin}}S_{drift}\left(\theta \right)\right) \end{array}$$

must be below the TV data estimate by a detection threshold of 4σ_−_ for at least six data points, and (iii) the difference between the TV data estimate and the initial drift estimate must not be a strictly increasing function of time after $t_{\max}^{0}$. These three criteria allow for the detection of the TR and further analysis of its characteristics [Section Transient Response (TR) Model].

**Figure 3:**
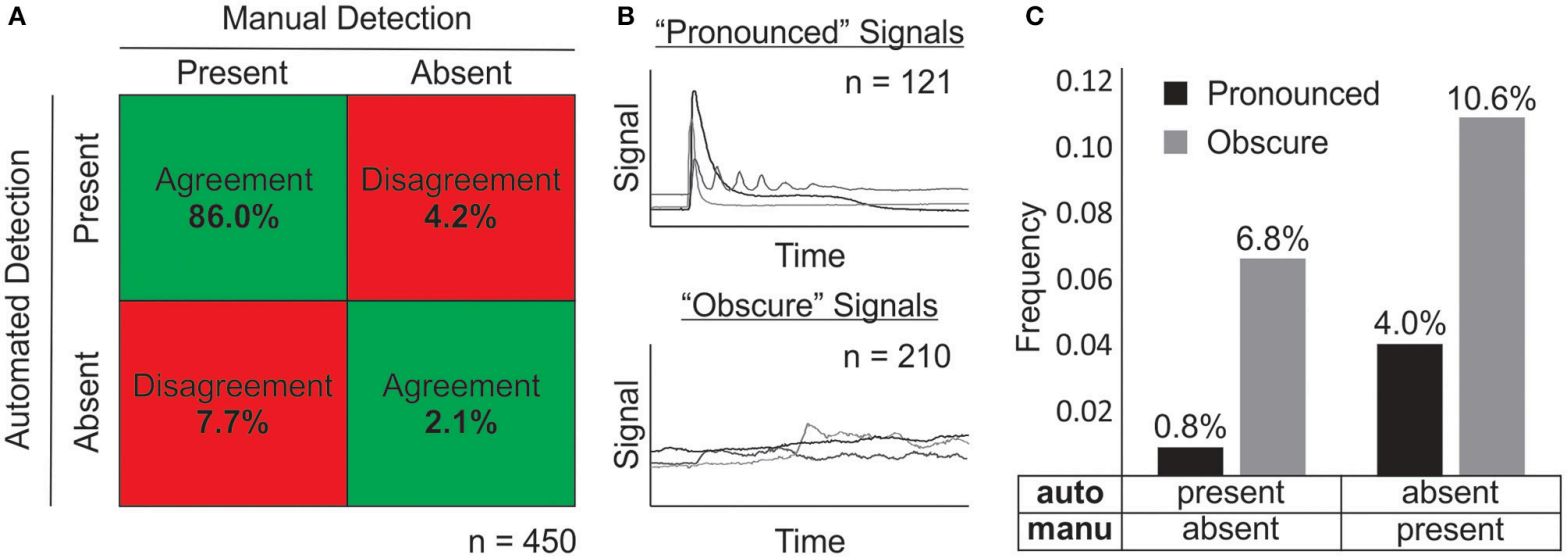
**Signal detection performance of algorithm. (A)** Signal detection analysis demonstrates relative selectivity and sensitivity of the algorithm as compared to user-mediated manual detection. “Agreement” refers to instances where the detection of a response was consistent between algorithm and manual methods. “Disagreement” refers to the contrary. “Present” indicates cases where a response was detected using the indicated method, while “absent” refers to cases where a response was not detected. **(B)** Representative time-series for pronounced (top) and obscure (bottom) signals. **(C)** Analysis of disagreement cases for pronounced and obscure signals. Automated detection, auto; manual detection, manu; sample size, n.

To evaluate the signal detection performance of the algorithm, 450 individual traces of ATP-induced calcium responses were used as a validation set. Manual results were then compared to automated detection of TRs to assess extent of agreement between the two methods. Classical signal detection nomenclature (i.e., true positive or negative and false positive or negative) was intentionally avoided due to lack of certainty in determining the true presence of TRs in more ambiguous cases. We found that the automated and manual methods agreed in detecting a TR in 88.1% of cases, and they disagreed in 11.9% of cases (Figure 3A). Further dissection of these results showed that 64.7% of disagreement arose from the algorithm reporting an absence of TR while visual evaluation suggested otherwise (Figure 3A), indicating that the algorithm has a tendency to be more conservative than user-mediated assessments.

To determine whether there were particular types of recordings that contributed to these disagreements, time-series traces were qualitatively divided into two groups: Clean signals with clearly defined responses were classified as “pronounced” (Figure 3B, top), and signals containing ambiguous signals with low signal to noise ratio or strong drift were classified as “obscure” (Figure 3B, bottom). The total frequency of disagreement was 3.6 times greater for obscure signals compared to those classified as pronounced (17.4 vs. 4.8%; Figure 3C). Regardless of the group, the algorithm signal detection remained more conservative compared to the manual method.

### Transient response (TR) model

Transient cellular responses are generally complex with multiple time scales and amplitudes. They may, in fact, exhibit prolonged MPRs superimposed on a more acute response (see Figure 2E). For these reasons, a complete characterization of all possible TRs is unlikely to be attainable. In order to remain as general as possible, we propose modeling TR as a continuously differentiable piece-wise defined function that first increases during the activation phase and subsequently decreases during the deactivation phase. Due to the large number of parameters required for such a description and the automated nature of our fitting procedure, we decompose the fitting of the whole TR into a sequential fitting of the activation phase alone followed by a fit of both phases simultaeously. This yields significantly more reliable results with faster convergence rates over a wide gamut of input data, because it allows for information obtained during preliminary simple fits to be used in a progressively more complex manner. In order to capture the complex fluorescence response generated by the spatially separated units in a live cell, we use a combination of Hill functions and quasi-linear functions generated by the integral of the corresponding Hill functions.

#### Response fitting

Following the least squares fitting of the activation data, we now seek to fit the entire recording (with the drift and TR) using a continuously differentiable function. A decreasing Hill Function is used to describe deactivation phase of the signal in a manner similar to Equation (11), as follows

$$\begin{array}{l} g_{de}\left(t;A_{de},\gamma ,n_{de}\right)=A_{de}\frac{\gamma^{n_{de}}}{t^{n_{de}}+\gamma^{n_{de}}}+m_{de}\overset{t}{\underset{0}{\int }}\frac{\gamma^{n_{de}}}{x^{n_{de}}+\gamma^{n_{de}}}dx, \end{array}$$

where *A*_*de*_ is the amplitude of the Hill function, *n*_*de*_ is the Hill coefficient, γ is the time at which the Hill function reaches its half maximum, and *m*_*de*_ is the slope of the quasi-linear function that accounts for the trend dominating at the beginning of deactivation. The time when the response switches to the deactivation function is denoted by *t*_*de*_. The parameter *m*_*de*_ is chosen such that the response function returns to zero by the end of the recording and is given by

$$\begin{array}{l} m_{de}=\frac{g_{act}\left(\ell_{act};A_{act},\beta ,n_{act},m_{act}\right)-A_{de}\frac{\gamma^{n_{de}}}{{\left(t_{N}\;-\;t_{de}\right)}^{n_{de}}\;+\;\gamma^{n_{de}}}}{\overset{t_{N}\;-\;t_{de}}{\underset{0}{\int }}\frac{\gamma^{n_{de}}}{x^{n_{de}}\;+\;\gamma^{n_{de}}}dx}, \end{array}$$

where ℓ_*act*_ = *t*_*de*_−*t*_*on*_ is the time duration of the activation phase of the response. If differentiability is not enforced at the point *t*_*de*_, where the two functions *g*_*act*_ and *g*_*de*_ meet, then the fitting may contain sharp edges indicative of unconverged solution. To solve this issue, the continuity of the first derivative of these two functions, particularly at *t*_*de*_, is achieved through a third-order Hermite spline (Traub, 1964) on the time interval [*t*_*de*_−ς_*act*_, *t*_*de*_+ς_*de*_], where ς_*act*_ and ς_*de*_ are two parameters that must satisfy ς_*act*_ < ℓ_*act*_ and ς_*de*_ < 2γ (see Data Sheet 2 in Supplementary Materials). The overall response model is thus given by

$$g_{resp}\left(t;\theta_{resp}\right)=\{\begin{array}{cc} g_{act}\left(t-t_{on};\theta_{resp}\right) & if\;t_{on}\leq t<t_{de}-\varsigma_{act}\\ p_{Hermite}\left(t-\left(t_{de}-\rho_{act}\right);\theta_{resp}\right) & if\;t_{de}-\varsigma_{act}\leq t<t_{de}+\varsigma_{de}\\ g_{de}\left(t-t_{de};\theta_{resp}\right) & if\;t_{de}+\varsigma_{de}\leq t \end{array},$$

where θ_*resp*_ = [θ_*act*_, *t*_*de*_, ς_*act*_, ς_*de*_, *A*_*de*_, γ, *n*_*de*_θ_*drift*_]. Given the response model, we define the global data model as

(13) $$G_{resp}\left(t;\theta_{resp},\theta_{drift}\right)=\{\begin{array}{cc} D\left(t;\theta_{resp}\right)+g_{resp}\left(t_{\text{i}};\theta_{resp}\right) & if\;t_{on}\leq t\\ D\left(t;\theta_{resp}\right) & otherwise \end{array},$$

and minimize the error function

(14) $$\begin{array}{l} S_{resp}\left(\theta_{resp}\right)=\phi \sum_{j}[\begin{array}{l} \phi {\left(F_{j}-G_{resp}\left(t_{j};\theta_{resp}\right)\right)}^{2}\\ \;+{\left({\hat{u}}_{j}-{\dot{G}}_{resp}\left(t_{j};\theta_{resp}\right)\right)}^{2} \end{array}\\ \;+\kappa \phi {\left(D_{2}\left(t_{k}-t_{2};a_{2},\tau_{2},m_{2}\right)\right)}^{2}]+\frac{\lambda }{\phi^{2}}S_{drift}\left(\theta_{drift}\right), \end{array}$$

to obtain the fitting, where κ is a parameter quantifying the apparent coherence between the drift and reponse models (*D* and *g*_*resp*_), given by

$$\kappa =\left|\frac{{\left(A\hat{u}\right)}_{N}-g_{act}\left(t_{max}-t_{on};A_{act},\beta ,n_{act},m_{act}\right)}{{\left(A\hat{u}\right)}_{N}-\underset{\text{i}\in {\text{i}}_{\text{act}}^{0},\ldots ,{\text{i}}_{\text{max}}}{\text{min}}{\left(A\hat{u}\right)}_{\text{i}}}\right|,$$

λ is a parameter defined by

$$\lambda =\frac{\underset{\text{i}>{\text{i}}_{\text{end}}^{0}}{\text{max}}\left|{\hat{u}}_{\text{i}}\right|}{\underset{\text{i}\in {\text{i}}_{\text{act}}^{0},\ldots ,{\text{i}}_{\text{max}}}{\text{max}}{\hat{u}}_{\text{i}}},$$

and the values for the parameters *A*_*act*_, *n*_*act*_, β, *m*_*act*_, and *t*_*on*_ are taken from the activation fitting. The first two terms in the sum of squares in Equation (14) are analgous to those in Equation (12), whereas the third term minimizes the AUC for the second drift function *D*_2_. By including the coefficient κ in this third term, however, allows *D*_2_ to become more significant when there is a large mismatch in the value of the baseline between $t_{{\text{i}}_{\text{act}}^{0}}$ and *t*_*N*_ (that cannot be explained by *d*_1_).

#### Activation parameter validation: t_onset_, t_10–90%_, and amplitude

**Figure 4:**
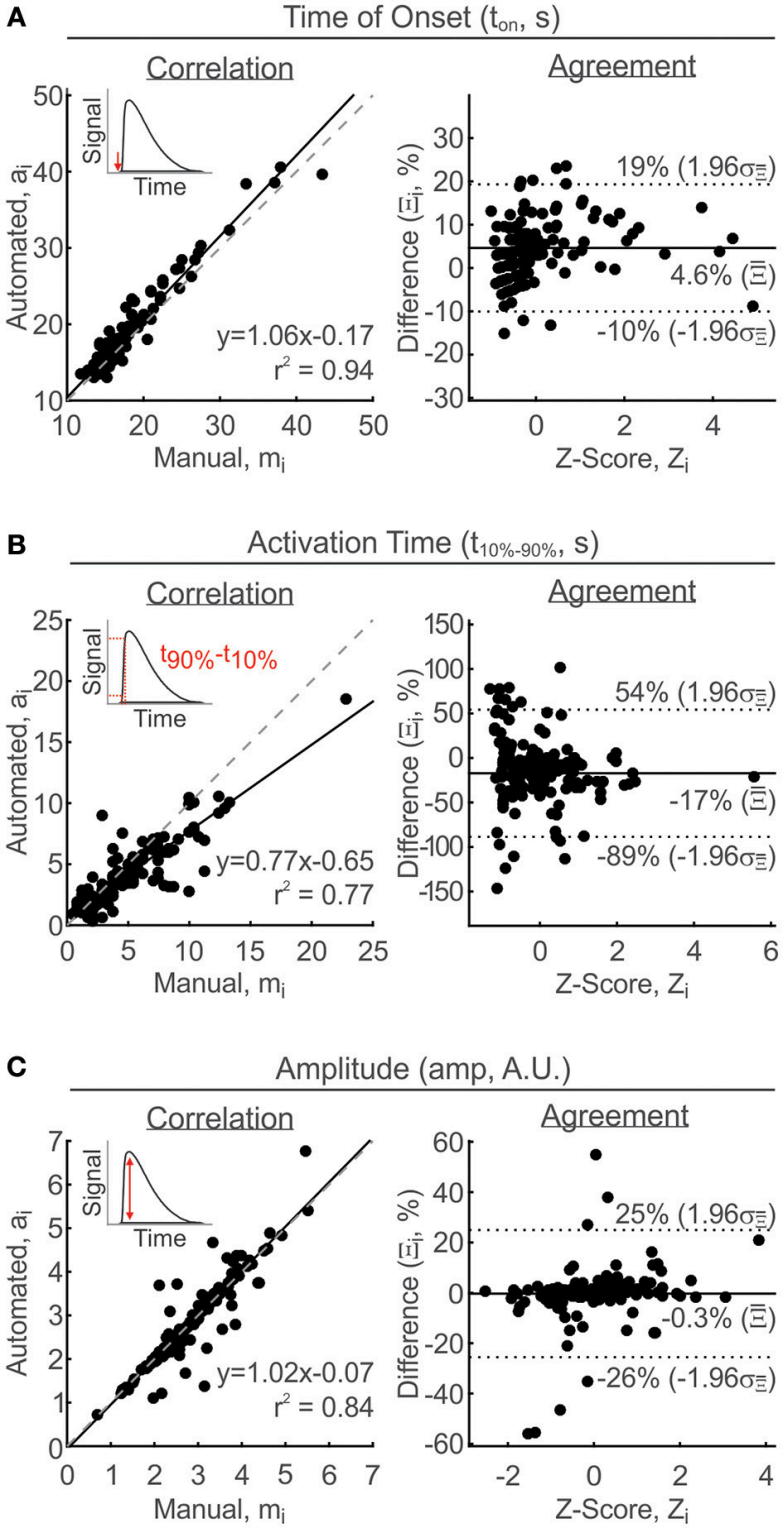
**Validation of activation parameter measurements**. Parameters were estimated manually and then compared with automated-estimated values using correlation analysis (left: solid line, linear regression line; broken line, line of equality) and Bland-Altman analysis (right, solid line: mean percent difference, $\bar{\Xi }$; broken line: limits of agreement, $\bar{\Xi }$ ±1.96 $\sigma_{\bar{\Xi }}$). **(A)** Time of onset, t_onset_. **(B)** Activation time, t_10–90%_. **(C)** Amplitude. Arbitrary units, A.U. Insets: Visual representations of parameters measured.

The three parameters, t_onset_, t_10–90%_, and amplitude, are considered together because they describe what happens at the activation phase of the TR, with no regard for the deactivation phase or MPR. The time at which a TR is discernable from baseline is defined as t_onset_ and is estimated directly as the parameter *t*_*on*_ of the response function *g*_*resp*_. There was a strong linear agreement between the manual and automated-estimates, with a linear slope of 1.06 and a correlation coefficient (*r*^2^) of 0.94 (Figure 4A, left). On average, automated estimates of t_onset_ were 4.6% greater than manual estimates, with limits of agreement ranging between −10 and 19% difference (Figure 4A, right). The t_10–90%_ is also estimated numerically from the response function *g*_*resp*_. Using the response function allows to overcome issues arising from the subsampling of rapid dynamics by numerically evaluating on a time grid 10 times finer than the input times. Manual estimation of this parameter is contingent upon accurate estimation of the baseline and peak occurrences, both of which present potential sources of error, particularly for noisy signals with drift. The relationship between manual and automated estimates had a linear slope of 0.77 and a correlation coefficient of 0.77 (Figure 4B, left). The higher degree of scatter away from the line of exact linear correlation is reflected by the wider Bland–Altman interval of agreement, ranging from −89 to 54% difference between manual and automated estimates. Overall, there was a −17% difference between all paired estimates of t_10–90%_, revealing that t_10–90%_ was manually overestimated compared to the automated estimates (Figure 4B, right). Amplitude estimates obtained by the manual and automated methods had a strong linear relationship with a slope of 1.02 and a correlation coefficient of 0.84 (Figure 4C, left). The limits of agreement, ranging from −26 to 25%, were narrow with a mean percent difference of −0.3% between all paired estimates of amplitude (Figure 4C, right).

#### TR parameter validation: AUC, FWHM, and τ_decay_

**Figure 5:**
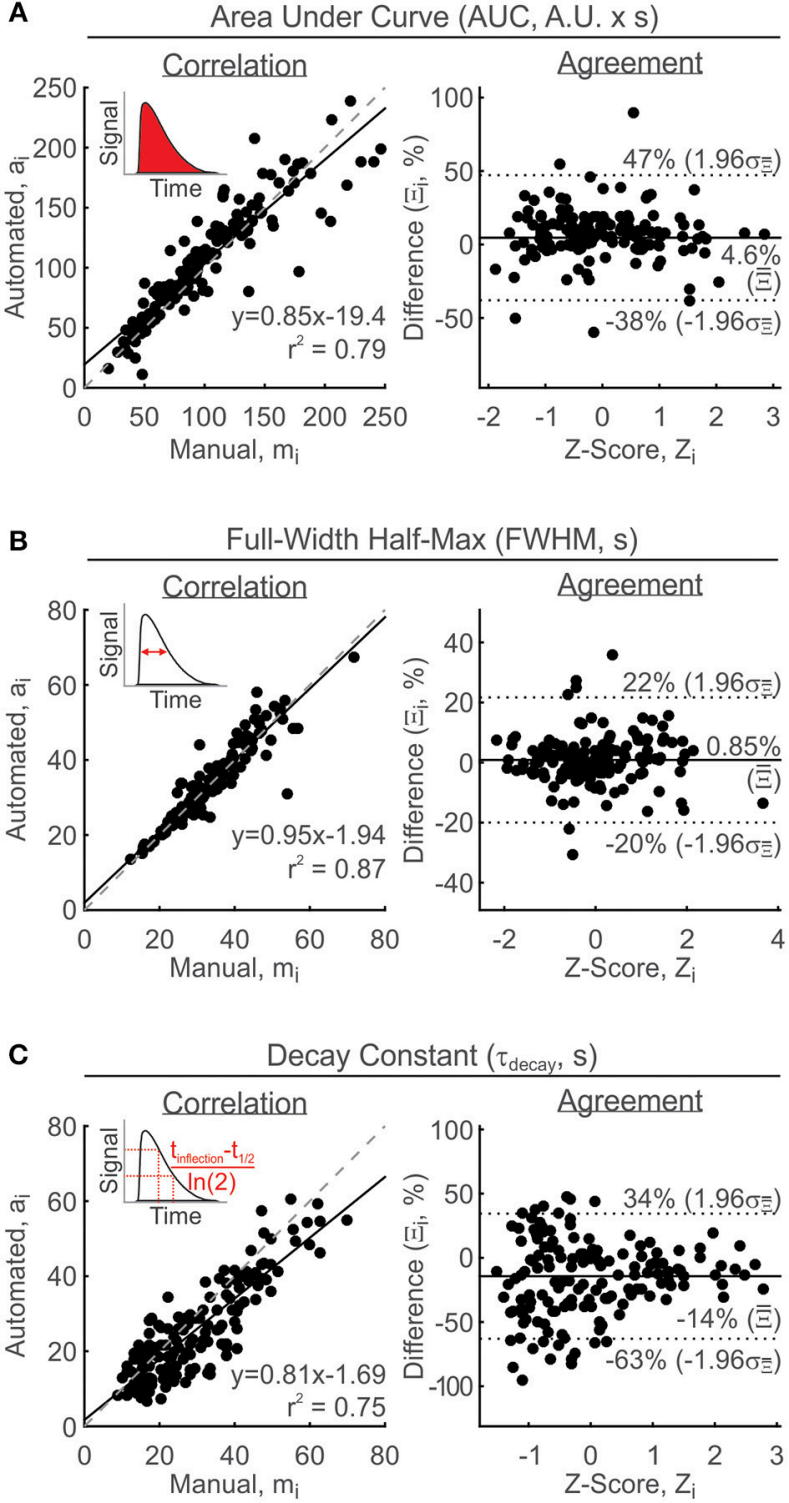
**Validation of TR parameter measurements**. Parameters were estimated manually and then compared with automated-estimated values using correlation analysis (left: solid line, linear regression line; broken line, line of equality) and Bland-Altman analysis (right: solid line, mean percent difference, $\bar{\Xi }$; broken line, limits of agreement, $\bar{\Xi }$ ±1.96 $\sigma_{\bar{\Xi }}$). **(A)** Area under curve, AUC. **(B)** Full-width half-max, FWHM. **(C)** Decay constant, τ_decay_. Arbitrary units, A.U. Insets: Visual representations of parameters measured.

Due to the inherent differences between TRs and MPRs and the approach taken with this algorithm, the AUC, FWHM, and t_decay_ are limited to describing TRs. Nevertheless, these parameters will be also reported in the presence of MPRs where they should be interpreted with the following considerations. (i) if a MPR is superimposed on a TR, the reported parameters describe the underlying TR, not the superimposed MPR. (ii) if TR presence is not detectable and MPR demonstrates a purely oscillatory response, the reported parameters characterize the first peak only. With these considerations in mind, manual evaluation of TR parameters was performed with a variety of signals, including MPRs. AUC estimates are manually determined using a geometric estimation of the area of a triangle whose vertices are at the start, peak, and end of the TR. Algorithmically, AUC was evaluated from the area under *g*_*resp*_, using the trapezoidal rule (Rice, 1973) implemented in MATLAB using “trapz().” Comparing the manual and automated estimates demonstrated a linear relationship with a slope of 0.85 and a correlation coefficient of 0.79 (Figure 5A, left). On average, automated estimates were 4.6% larger than manual estimates with an interval of agreement ranging between −38 and 47% difference (Figure 5A, right). Considering the geometric-approach used for manual-estimation of AUC values, it is reasonable to assume that the error arose from manual limitations.

Due to the difficulty in determining the precise time at which the signal returns to its former baseline, it would be challenging to manually describe the duration and decay characteristics of the response. Incomplete recordings and background drift are largely responsible for generating such behavior. The full-width half-max (FWHM) is defined as the time elapsed between the two half-max coordinates of a peak. Our analysis of FWHM revealed that the linear relationship between manual and automated-estimates was strong, with a correlation coefficient of 0.87 and slope of 0.95 (Figure 5B, left). The Bland Altman analysis demonstrated that the agreement interval ranged from −20 to 22% difference with a mean percent difference of 0.85% between all paired estimates (Figure 5B, right).

Finally, to manually estimate the decay constant, the time of the inflection point (ρ) is visually estimated and the general trend of the data following ρ is represented by a mono-exponential decay. The decay constant is then determined by the time it takes for the signal to reduce to approximately 37% of its initial value (1/e). Algorithmically, the time ρ is determined by solving for the inflection point in the deactivation function, given by

$$\begin{array}{l} \rho =t_{de}+\gamma {\left(\frac{n_{de}-1}{n_{de}+1}\right)}^{1/n_{de}}. \end{array}$$

The data following ρ is then fit to a mono-exponential decay function using least squares, to determine the time constant of decay. The slope of the linear agreement between manual and automated-estimates was 0.81 and the correlation coefficient was 0.75 (Figure 5C, left). The Bland Altman analysis revealed a strong systematic bias of −14% difference, with an agreement range of −63 to 34% difference, signifying that manual efforts to estimate the decay constant consistently overshot the values reported by the algorithm (Figure 5C, right).

### Multi-peaked responses

To isolate the characteristic parameters of MPRs that are frequently superimposed on TRs (Figure 2D), the TR model *g*_*resp*_ must be refined to serve as a non-stationary baseline around which the MPR will oscillate. This refinement is necessary because it is often the case that the TR model *g*_*resp*_ will produce sub-optimal fittings where the data deviates significantly from the TR fitting. Therefore, to accurately characterize the TR and quantify the properties of truly oscillatory MPRs, it is first necessary to adapt the least squares fitting procedure of Section Response Fitting to remove the effects of data points not well represented by *G*_*resp*_. This is done by first identifying large deviations representing MPRs from the *G*_*resp*_ -fitting (obtained by minimizing Equation 14), and then reweighing the sum of squares in Equation (14) to remove the influence of those deviations from the fits. We subsequently perform a secondary fitting of *G*_*resp*_ to determine more accurately the baseline, delineating the TR, where the MPR-associated deviations originate from. Finally, we analyze the MPRs by determining whether they represent oscillations and, if so, quantify their properties.

To identify these MPR-associated deviations from the newly defined baseline, we first employ the MATLAB “findpeaks()” function. This finds the peaks and troughs of the significant deviations in the TV data estimate, *Aû*(*t*), from the TR estimate, $G_{resp}\left(t;{\hat{\theta }}_{resp}\right)$, truncated at its half-maximums, where ${\hat{\theta }}_{resp}$ is the optimal parameter set obtained from minimizing Equation (14). This truncation permits for the possibility that the onset of TR coincides with the first peak of the MPR-associated deviations. The implementation of peak-finding algorithm, on the other hand, allows for the specification of minimum heights and timing between peaks, set to be 6σ_−_ and 5 s, respectively. The algorithm yields the heights (*E*^*peak*^, *E*^*trough*^), the FWHM (ξ^*peak*^, ξ^*trough*^), and the times (${\tilde{t}}^{peak}$, ${\tilde{t}}^{trough}$) of significant peaks and troughs of the deviations, respectively (see Figure 2D). In total, there are Ñ = *N*_*peak*_+*N*_*trough*_ of these deviations, including *N*_*peak*_ peaks and *N*_*trough*_ troughs. If Ñ ≤ 2, then the only deviation in the signal is the TR and the algorithm can terminate. Without prior knowledge of the nature of the MPR-associated deviations, it is very difficult to determine whether they result from trends which are above, below, or symmetric to the TR. To resolve this issue, we assume that the estimated baseline from Section Response Fitting underlies the signal in the absence of deviations. To incorporate this assumption algorithmically, we define two bias parameters based on the relative heights of the first peak and trough, as follows

$$\begin{array}{lll} o_{peak} & = & \exp\left[-{\left(\frac{E_{1}^{peak}}{E_{1}^{trough}}\right)}^{4}\right]\text{ and}\\ o_{trough} & = & \exp\left[-{\left(\frac{E_{1}^{trough}}{E_{1}^{peak}}\right)}^{4}\right]. \end{array}$$

These quantities are then used to calculate weighting functions for the data based on the properties of peaks

$$\begin{array}{l} \pi_{\text{i}}^{peak}=\overset{N_{peak}}{\underset{r=1}{\sum }}o_{peak}\exp\left(-\phi {\left(\frac{o_{peak}\left(t_{\text{i}}-{\tilde{t}}_{r}^{peak}\right)}{\xi_{r}^{peak}}\right)}^{2}\right)\\ +\;\left(1-o_{peak}\right)\left(1-\exp\left(-\phi {\left(\frac{o_{peak}\left(t_{\text{i}}-{\tilde{t}}_{r}^{peak}\right)}{\xi_{r}^{peak}}\right)}^{2}\right)\right) \end{array}$$

and troughs

$$\begin{array}{ll} \pi_{\text{i}}^{trough}\\ = & \overset{N_{trough}}{\underset{r=1}{\sum }}o_{trough}\exp\left(-\phi {\left(\frac{o_{trough}\left(t_{\text{i}}-{\tilde{t}}_{r}^{trough}\right)}{\xi_{r}^{trough}}\right)}^{2}\right)\\ +\;\left(1-o_{trough}\right)\left(1-\exp\left(-\phi {\left(\frac{o_{trough}\left(t_{\text{i}}-{\tilde{t}}_{r}^{trough}\right)}{\xi_{r}^{trough}}\right)}^{2}\right)\right). \end{array}$$

The weighting functions $\pi_{\text{i}}^{peak},\pi_{\text{i}}^{trough}$ quantify the relative reliability of the data around each deviation based on how close it is to the fitting function *G*_*resp*_ and on its duration. We can also assess the reliability of each time point of the recording (including the TR, the drift, and any MPR-associated deviations present) by how well its derivative matches ${Ġ}_{resp}\left(t;{\hat{\theta }}_{resp}\right)$. This is done using another weighting function, defined by

$$\begin{array}{l} \Gamma_{\text{i}}=\exp\left(-{\left(\frac{{\hat{u}}_{\text{i}}-{Ġ}_{resp}\left(t;{\hat{\theta }}_{resp}\right)}{2std\left({\hat{u}}_{\text{i}}-{Ġ}_{resp}\left(t;{\hat{\theta }}_{resp}\right)\right)}\right)}^{2}\right). \end{array}$$

We combine these weighting functions using the criterion that for a data point to be reliable, it must have either a large value of π^*peak*^ or π^*trough*^, and a large value of Γ. It is implemented in the weighting function Ω, as follows

$$\begin{array}{l} \Omega_{\text{i}}=\Gamma_{\text{i}}\left(\pi_{\text{i}}^{peak}+\pi_{\text{i}}^{trough}-\underset{\text{i}}{\min}\left(\pi_{\text{i}}^{peak}\right)-\underset{\text{i}}{\min}\left(\pi_{\text{i}}^{trough}\right)\right). \end{array}$$

With Ω_i_, we can fit the TR reliably in the presence of significant deviations from the model of Equation (13). This is done by minimizing the error function

(15) $$\begin{array}{l} S_{resp}^{\Omega }\left(\theta_{resp}\right)=\phi \underset{j}{\sum }\left[\begin{array}{l} \phi \Omega_{j}^{2}{\left(F_{j}-G_{resp}\left(t_{j};\theta_{resp}\right)\right)}^{2}\\ \;+\Omega_{j}^{2}{\left({\hat{u}}_{j}-{Ġ}_{resp}\left(t_{j};\theta_{resp}\right)\right)}^{2}\\ \;+\;\kappa \phi {\left(D_{2}\left(t_{k}-t_{2};a_{2},\tau_{2},m_{2}\right)\right)}^{2} \end{array}\right]\\ +\;\frac{\lambda }{\phi^{2}}S_{drift}\left(\theta_{drift}\right). \end{array}$$

#### Identifying coherent oscillations

Not all MPRs correspond to periodic oscillations (Thurley et al., 2014). To address this, the algorithm reports two sets of MPR parameters, the first to describe all the peaks detected within a MPR, and the second to describe the subset of coherent peaks present within the same MPR. This section focuses on how the subset of coherent oscillatory peaks is identified. We use a clustering algorithm which is an unsupervised learning technique that enables for the identification of natural groupings or patterns with a defined data set. By minimizing Equation (15), we obtain the most reliable estimate of the TR (specified by the model *G*_*resp*_ and its optimal parameter set ${\hat{\theta }}_{resp}^{\Omega }$), which we take to be the baseline of the MPR-associated deviations. Given the estimate $G_{resp}\left(t;\theta_{resp}^{\Omega }\right)$, we repeat the peak finding steps detailed in Section Multi-Peaked Responses. To determine whether or not the detected deviations represent oscillations, we use a Gaussian mixture model clustering technique. It groups together (in clusters) peaks and troughs with comparable periods, *T* (determined by the difference between two consecutive peak or trough times; i.e., ${\tilde{t}}^{peak}$ or ${\tilde{t}}^{trough}$) and FWHM, ξ. Two adjacent deviations are deemed to be coherent oscillations if they are grouped in the same cluster. In situations where the period or FWHM are modulated throughout time, Gaussian clustering technique may not be able to cluster all coherent oscillations adequately. We therefore process clusters by defining period- and FWHM-trends for all coherent oscillations. If this trend can accurately predict the period and FWHM of the first deviation of an adjacent cluster, then both clusters are deemed to form a set of coherent oscillations. This is repeated for all pairs of adjacent clusters, progressively updating the set of coherent oscillations with those previously deemed incoherent at prior steps.

This procedure use the Expectation Maximization (EM) clustering algorithm (McLachlan and Peel, 2005) to cluster the period and FWHM of a potential oscillatory MPRs, and to determine the optimal number of clusters using gap statistic (Tibshirani et al., 2001). It allows for a reliable separation of oscillatory data from recording artifacts or non-oscillatory MPRs with visually different properties. It also yields a set of ${\hat{N}}_{peak}$ peaks (${\hat{N}}_{trough}$ troughs) occurring at times ${\hat{t}}^{peak}$ (${\hat{t}}^{trough}$), which together represent coherent oscillations. Having determined the properties of the individual features making up the oscillations, they can be used to quantify the properties of the oscillations.

#### Characterizing oscillatory parameters

In order for the algorithm to report the oscillatory properties of a signal, the MPR-associated deviations must satisfy Ñ > 2, for the set of MPR parameters describing all detected peaks. If these deviations form a coherent set of oscillations, a second set of MPR parameters characterizing this coherent oscillatory behavior is also reported. In both instances, the following parameters will be reported: the number of oscillations (*N*_*osc*_), the average magnitude of the oscillations (defined as *E*^*peak*^ + *E*^*trough*^), the average period of oscillation (*T*), the standard deviation of the periodicity (σ_*T*_), the total time for which the oscillations persist (defined as $\ell_{osc}={\hat{t}}_{{\hat{N}}_{peak}}^{peak}-{\hat{t}}_{1}^{peak}$), and the mean duty cycle parameter (given by ξ^*peak*^/*T*) (Smedler and Uhlén, 2014).

#### MPR parameter validation: N_osc_, E, T, *l*_osc_, and ξ^peak^/T

**Figure 6:**
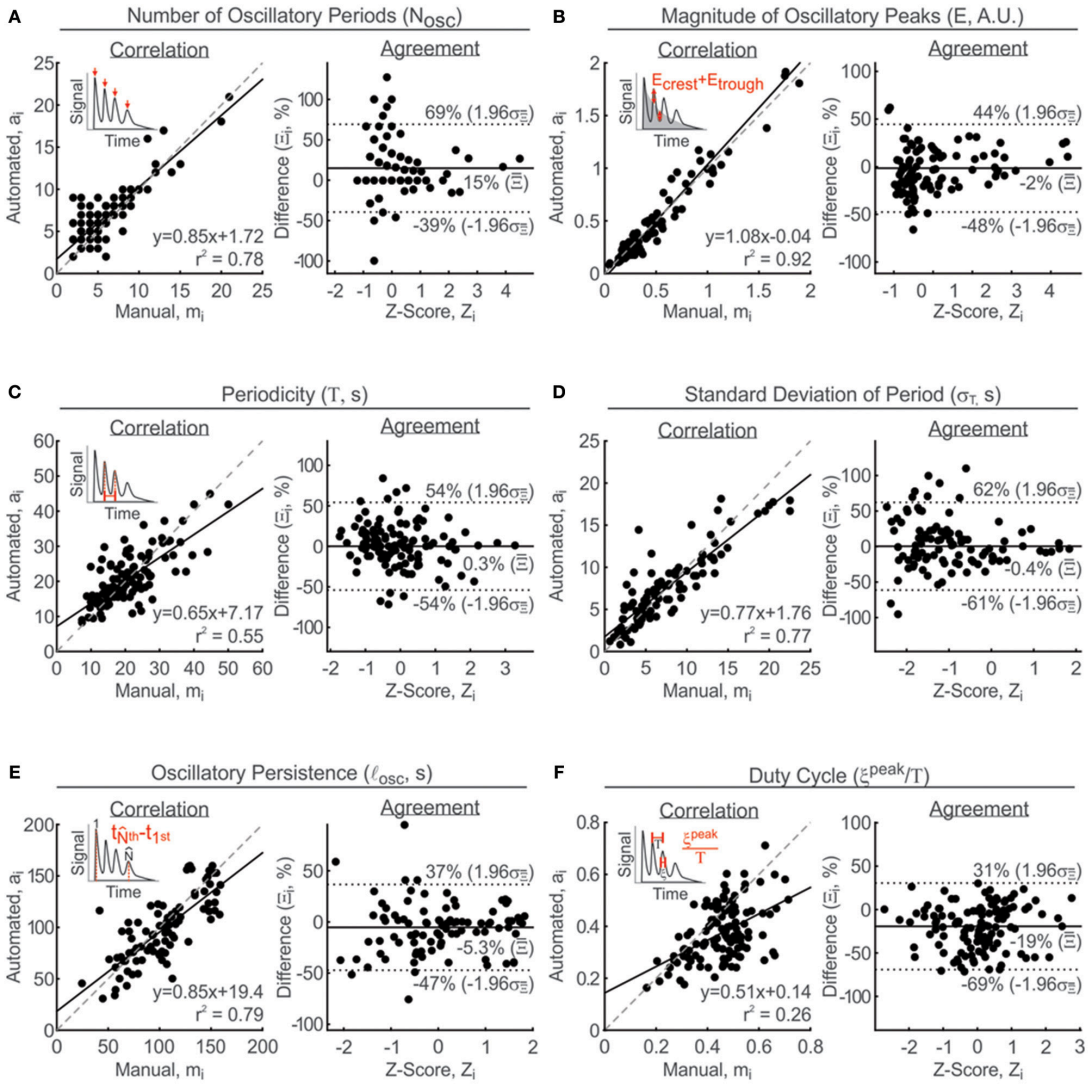
**Validation of MPR parameter measurements**. Parameters were estimated manually and then compared with automated-estimated values using correlation analysis (left: solid line, linear regression line; broken line, line of equality) and Bland-Altman analysis (right: solid line, mean percent difference, $\bar{\Xi }$; broken line, limits of agreement, $\bar{\Xi }$ ±1.96 $\sigma_{\bar{\Xi }}$). **(A)** Number of oscillatory periods N_osc_. **(B)** Magnitude of oscillatory peaks, E. **(C)** Periodicity, T. **(D)** Standard Deviation of Period, σ_T_. **(E)** Oscillatory persistence, *l*_osc_. **(F)** Duty cycle, ξ^peak^/T. Arbitrary Units, A.U. Insets: Visual representations of parameters measured.

Manual estimates of N_osc_ is determined by counting the number of discernable peaks within the signal. The slope of correlation was 0.85 with an *r*^2^-score of 0.78 (Figure 6A, left). The mean difference between manual and automated estimates was 15% with an interval of agreement ranging from −39 to 69% (Figure 6A, right). The peak magnitude of the oscillations, E, is manually estimated by the mean change between peak maxima and their subsequent trough minima, after correcting for a non-stationary baseline that is often a consequence of a concurrent TR. For most signals the non-stationary baseline can be manually estimated to be linear. However, there are a few cases where an estimate of an exponential baseline is required. The correlation between manual and automated estimates was relatively strong, with an *r*^2^-value of 0.92 and a slope of 1.08 (Figure 6B, left). The agreement analysis on the other hand revealed relatively no bias, with a mean difference of −2% and limit of agreement ranging from −48 to 44% difference (Figure 6B, right). The periodicity is manually estimated by the average time between adjacent peaks. The linear relationship was slightly weaker with a *r*^2^-value of 0.55 and slope of 0.65 (Figure 6C, left). The mean difference between manual and automated estimates was negligible, at only 0.3%, indicating an absence systematic bias, and the limits of agreement spanned from −54 to 54% difference (Figure 6C, right). The standard deviation of periodicity was obtained from the same set of periods used to estimate the mean period. The linear slope was 0.77 and the *r*^2^-value was 0.77 (Figure 6D, left). Similar to periodicity, the mean difference for the standard deviation of periodicity was a negligible −0.4%, with limits of agreement ranging from −61 to 62% difference (Figure 6D, right). Oscillatory persistence is chosen to describe how long oscillations are sustained within a given recording, and is estimated as the elapsed time between the first and last discernable peaks in the MPR. The correlation between manual and automated estimates was supported by a *r*^2^-value of 0.79 and slope of 0.85 (Figure 6E, left). The mean difference between paired estimates was only −5.3% with a limit of agreement between −47 and 37% difference (Figure 6E, right). Finally, the duty cycle is manually estimated by the ratio between ξ^peak^ and T. ξ^peak^ is manually determined by the mean FWHM of individual oscillatory peaks and the same T value obtained above is used to calculate x^peak^ /T. The linear relationship between manual and automated estimated of ξ^peak^ /T was decidedly weak with a slope of 0.51 and *r*^2^ of 0.26 (Figure 6F, left). The Bland Altman analysis, however, suggests that there was a systematic bias that could explain the poorer correlation results. The mean difference between manual and automated estimated was −19% with a limit of agreement between −69 and 31% (Figure 6F, right).

The MPR parameter validation described above focuses on the set of parameter estimates describing all the peaks in the MPR, rather than the subset of coherent oscillations. This is because manual detection of each peak in the MPR is less subjective than detecting only the coherent peaks in the MPR. Since the algorithm sub-selects the coherent oscillatory peaks from the initial set of identified deviations, the performance reported for the characterization of all peaks extends to the subset of coherent oscillations. Furthermore, as expected, the standard deviation of the periodicity is consistently lower for coherent oscillations when compare to the σ_T_ reported for all peaks in the same MPR (i.e., more regular periodicities result in lower standard deviations).

To ensure confidence in the reported MPR parameters, users of this algorithm are urged to visually verify the quality of the signal fittings to determine whether the algorithm is characterizing their peaks of interest, as these may not always coincide with the most prevalent oscillatory component of the signal (Thurley et al., 2014). Furthermore, N_osc_ reported for all peaks and coherent peaks can be compared to be aware of how many peaks were omitted during the clustering step. Collectively, the information reported for MPRs is sufficient for the informed analysis of a diverse selection of MPRs, including those that exhibit coherent oscillations and those that do not.

### Application to pathophysiology

In the context of bone physiology, the deleterious consequences of disrupting extracellular nucleotide-mediated cross talk have been highlighted by the emergence of P2 receptor knockout mouse models (Lenertz et al., 2015). P2 receptors are particularly sensitive to changes in the extracellular milieu. Consequently, P2 receptor pathophysiology is often coupled to events that influence the extracellular composition, thereby compromising processes regulated by the P2 receptor network. In particular, changes in extracellular pH alter P2 receptor function (King et al., 1997; Gerevich et al., 2007; Wildman, 2009; Langfelder et al., 2015). Such conditions arise from pathological acidosis that is commonly caused by systemic acid-base disturbances, such as metabolic or respiratory acidosis (Krieger et al., 2004; Miller, 2012; Berend et al., 2014). More localized acidifications can also be associated with tumors (Martin and Jain, 1994; Kato et al., 2013). Since the skeleton is a common metastatic site for cancer, and participates in systemic buffering of protons, the effect of acidosis on the skeletal system is of particular interest. On the cellular level, acidosis promotes the activation of osteoclasts, resulting in elevated bone resorption which manifests itself in osteoporotic phenotype (Bushinsky and Frick, 2000; Krieger et al., 2003; Ahn et al., 2012; Gasser et al., 2014). However, it remains unclear whether the P2 receptor network plays a direct role in this cascade of events. The most immediate influence of acidosis on the P2-receptor network can be studied at the level of the [Ca^2+^]_i_ response evoked immediately upon application of a purinergic agonist.

**Figure 7:**
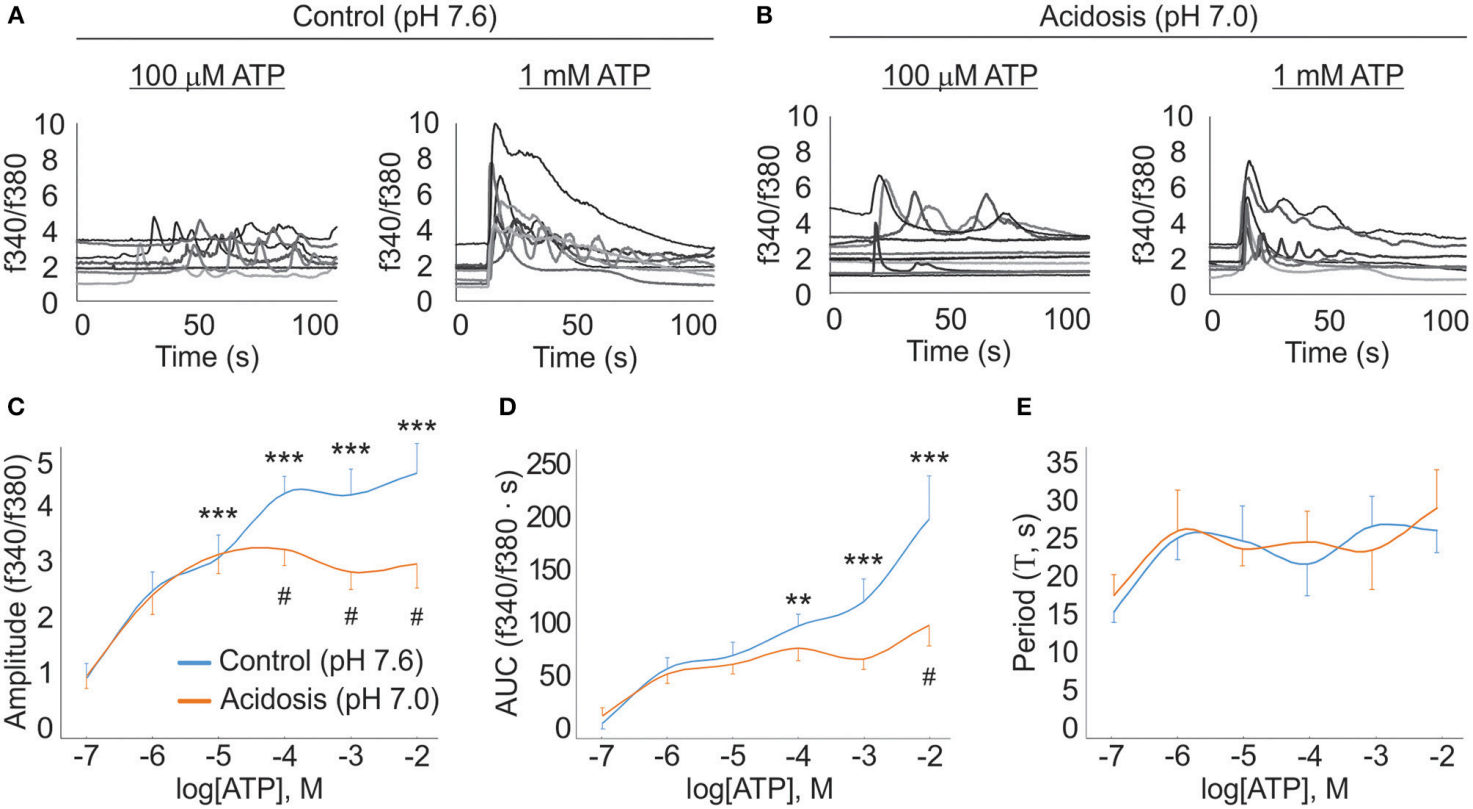
**Algorithm application in characterization of pathological states**. ATP (100 nM–10 mM) was applied to Fura-2 loaded osteoclast precursors, under control (pH 7.6) and acidosis (pH 7.0) conditions, and [Ca^2+^]_i_ responses were recorded. Algorithm was used to obtain estimates for amplitude, AUC and periodicity. **(A)** Representative [Ca^2+^]_i_ response traces for 100 μM and 1 mM ATP under control conditions. **(B)** Representative [Ca^2+^]_i_ response traces for 100 μM and 1 mM ATP under acidosis conditions. **(C)** Amplitude dose-response curves. **(D)** AUC dose-response curves. **(E)** Period dose-response curves. For **(C–E)**, data are mean ± S.E.M. The effect of ATP under control conditions was examined using one-way ANOVA. The effect of acidosis on ATP-mediated responses was examined using two-way ANOVA. The Bonferroni test was used for post hoc multi-comparison analysis; ^**^p < 0.01; ^***^p < 0.001 indicate significant difference compared to the response to the lowest ATP concentration; ^#^*p* < 0.05 indicates significant difference between responses to the same [ATP] in control and acidosis conditions.

We investigated the effect of acidosis on ATP-mediated [Ca^2+^]_i_ responses in bone-marrow derived osteoclast precursors to demonstrate the applicability of the developed algorithm. The application of ATP (100 nM to 10 mM) to the fura2-loaded osteoclast precursors evoked a [Ca^2+^]_i_ TR in a dose-dependent manner in control and acidosis conditions (Figures 7A,B). The response amplitudes under acidic conditions were virtually indistinguishable from the control for ATP concentrations up to 10 μM. However, above this threshold concentration, the amplitude of the control responses continued to increase with rising concentrations of ATP, while [Ca^2+^]_i_ responses under acidic conditions plateaued at 10 μM (Figure 7C). With respect to the AUC of the [Ca^2+^]_i_ responses, the observed differences between the two conditions were more gradual with a diverging trend beginning as low as 1 μM ATP and becoming more prominent at high ATP (Figure 7D). Finally, acidosis was found to have no significant effect on the periodicity of the oscillatory responses (Figure 7E).

These findings support that acidosis, while having no effect on ATP-mediated [Ca^2+^]_i_ responses at lower ATP concentration, significantly attenuates the magnitude of [Ca^2+^]_i_ transients responding to higher ATP concentrations (>10 μM ATP). Within the limited scope of this study that is focused on the development of a data analysis algorithm, we can only hypothesize on the mechanism by which these differences arise. One possibility is that the rise in extracellular [H^+^] has a significant influence on the electro-chemical gradient across the cellular membrane, which may consequently alter the extent of calcium flux across certain ionotropic P2X receptors. Since the oscillations are commonly driven by inositol triphosphate-mediated release of calcium from internal calcium stores (i.e., isolated from extracellular [H^+^]), it may explain why the oscillatory behavior is not affected by acidosis. Alternatively, there may exist a subset of P2 receptors that are sensitive to fluctuations in extracellular [H^+^], while P2-receptors involved in oscillatory behavior and/or responses to lower ATP concentrations (≤10 μM) are resilient to such changes. Regardless of the underlying mechanism, these results highlight that the P2 receptor network can be differentially modulated by extracellular pH.

## Conclusions

This paper presents an autonomous signal-processing algorithm capable of robustly removing signal-contaminating noise and delineating the various components seen in a calcium response, including non-stationary drift, TRs, and MPRs (possibly caused by flickers, puffs, and sparks) sampled with at least twice the Nyquist frequency. By fitting piece-wise defined model functions to data, the algorithm also extracts estimates for the parameters that are relevant to the characterization of cellular transient dynamics. Any time-series recordings can be used as an input for the algorithm, provided that they resemble a single or multi-peak transient response. As demonstrated in the validation process, manual estimation of certain parameters has an inherent degree of subjectivity and measurement error associated with it. In particular, the manual evaluations of AUC values, decay constant and the time of onset, as well as most of the MPR-parameter values, were found to rely on subjective estimates and thus lacked true accuracy and consistency. Because of such limitation in the validation method, manual-estimates are to be recognized as representative estimates, rather than accurate values for these parameters. Consequently, validation method applied here should be considered as a comparison against imperfect estimates.

Nevertheless, our analysis of the automated method has verified that the algorithm performs within acceptable margins of agreement when compared to manual analysis. Regarding the response detection capabilities, the algorithm behaves conservatively compared to manual assessments, especially when presented with low-magnitude TRs or ambiguous response signals. Most importantly, our algorithm has been validated against experimental [Ca^2+^]_i_ recording data, rather than simulated data, ensuring that the method is capable of handling variations in drift and noise that realistically reflect signal contaminations of experimental data acquisition. We have demonstrated that this automated methodology is effective in analyzing empirical data, providing quantitative insights about them and identifying differences between them.

A particularly unique feature of this algorithm is its capacity to characterize the magnitude and temporal characteristics of MPRs exhibiting stochastic and deterministic behavior. It is well established that a diverse amount of biochemical processes can be amplitude- and/or frequency-modulated (Adachi et al., 1999; Micali et al., 2015). To analyse such oscillatory data, the fast Fourier transform (FFT) is commonly used, which allows for the conversion of a signal from its time domain, into the frequency domain. Unfortunately, the variance in the frequency domain is proportional to the number of repetitive components in the time-domain. Therefore, if the oscillatory signals present few repetitive components then reliable resolution of the true periodicity of the signal is unachievable. To circumvent the limitations inherent to FFT, we apply the MATLAB “findpeaks()” function to identify peaks of interest. To isolate underlying coherent oscillations that are often present, we applied a clustering method. This is based on the principle of clustering deviations from baseline according to their temporal offset and respective FWHM. The advantage of this approach is that it allows for the reliable detection of periodic peaks, even in the presence of stochastic discharges, as is often the case in experimental recordings. Secondly, comparison of the set of MPR parameters for all peaks and subset of coherent peaks allows users to quantify the extent of stochastic activity within MPRs. Alternatively, the relationship between mean and standard deviation of periodicity in a MPR has been previously used to reveal the contribution of stochastic processes to the periodicity (Thurley et al., 2014). We anticipate this methodology will contribute to the comprehensive analysis of diverse MPRs.

Calcium signaling is by no means unique to the P2-receptor network, but rather represents the most ubiquitous and versatile messenger found in biological systems. All kinds of extracellular signals exploit calcium as a secondary messenger, including P2 agonists (i.e., ATP, ADP, UTP, and UDP), endothelin-1, oxotremorine-M, norephinephrine, thrombin, PDGF, bombensin (Balla et al., 1991; Palmer, 1994; Burnstock, 2004). The universal involvement of calcium ranges from basic physiological processes such as muscle contraction, neuronal discharge and pancreatic secretion, to early development events including mammalian egg fertilization and embryonic pattern formation (Berridge et al., 2000). Calcium signaling is also known to be impaired in various pathological states, as suggested for metabolic acidosis in this study, chronic renal failure (Massry et al., 1995), Alzheimer's (Brawek et al., 2014), Diabetes (Chen et al., 2015), and zinc deficiency (O'Dell and Browning, 2013). However, despite all that we know about calcium's role in biological processes, there remains ongoing debate on how calcium signals robustly encode information while still exhibiting a large degree of heterogeneity within and between various cellular populations. Many theories have been proposed to establish how information can be encoded. Some of these involve encoding information on the basis of calcium binding cooperativity (Larsen et al., 2004), amplitude and frequency modulation (De Pitta et al., 2009), changes in spike time variation (Thurley et al., 2014), and signal integration (Hannanta-anan and Chow, 2016). In order to reconcile these theories and establish a universal syntax for calcium-encoded information, tools such as this algorithm will aid in the large-scale analysis of experimental data sets required for the validation of mathematical models.

The consideration of signaling nuances that are specifically found in physiological signals, but may or may not be present in non-biological signals, was a critical step in the development of this algorithm. As demonstrated in this study, physiological signals were decomposed into their elementary components and mathematically generalized to enable for the computational reconstruction of a diverse range of signature forms. In doing so, we were able to provide a foundation for further modeling of the nonlinear multi-parametric physiological signals. This study demonstrates that the accurate description of complex physiological signals is non-trivial, but rather an extensive mathematical undertaking. Therefore, we believe that, beyond serving the purpose of a signal-processing tool, this algorithm will also contribute to future efforts to modeling physiological signals.

In summary, we have detailed an open-source MATLAB algorithm intended to facilitate the analysis of time-series recordings. With minimal user-input required, this tool dramatically decreases analysis time and ensures consistency in parameter characterization of complex physiological signals. This algorithm is capable of handling noise and drift and robustly characterizes the magnitude and kinetics of dynamic processes, outputting the amplitude, time of onset (t_onset_), activation time (t_10–90%_), full-width half-max (FWHM), AUC, and decay constant (τ_decay_). In the presence of MPR, six additional parameters are characterized which include number of oscillations (N_osc_), magnitude of oscillatory peaks (E), periodicity (T), standard deviation of periodicity (σ_T_), oscillatory persistence (l_osc_), and the duty cycle (ξ^peak^/T). This algorithm is not limited to any specific data-type, but [Ca^2+^]_i_ recordings represent an obvious application. In addition to calcium imaging, other imaging modalities such as adapted fluorescence resonance energy transfer (FRET) biosensors, real-time bioluminescence and voltage and current measurements can generate time-series data for which characterization of signal magnitude and kinetics can provide valuable information. As data acquisition becomes more efficient and data sets become increasingly complex, automated analysis will serve as an essential tool for conducting basic research and clinical screening.

## Author contributions

Study conception and design: LM, NM, SK, AK. Algorithm development: LM, AK. Acquisition of data: NM. Analysis and interpretation of data: LM, NM. Drafting of Manuscript: LM, NM, SK, AK. All authors contributed to the critical revision of manuscript and approved the final version to be published.

### Conflict of interest statement

The authors declare that the research was conducted in the absence of any commercial or financial relationships that could be construed as a potential conflict of interest.

## Abbreviations

ADP: Adenosine diphosphate
ATP: Adenosine triphosphate
AUC: Area under curve
[Ca^2+^]_i_: Cytosolic free calcium concentration
D(t): Global drift model
E: Mean peak magnitude of MPR
FWHM: Full-width half-max
F(t): Measured fluorescent signal
*l*_osc_: Oscillatory persistence
MPR: Multi-peak response
N_osc_: Number of peaks in MPR
ξ^peak^: Width of oscillatory peak
P2: Purinergic
σ_T_: Standard deviation of periodicity
T: Period of oscillation
t_10–90%_: Activation time
τ_decay_: Decay constant
t_onset_: Time of response onset
TR: Transient response
TV: Total-variational.
