# Supplementary figures and images for: Systematic Characterization of Dynamic Parameters of Intracellular Calcium Signals
Source: Front Physiol. 2016 Nov 10;7:525. doi: 10.3389/fphys.2016.00525 (PMC5102910; doi:10.3389/fphys.2016.00525)

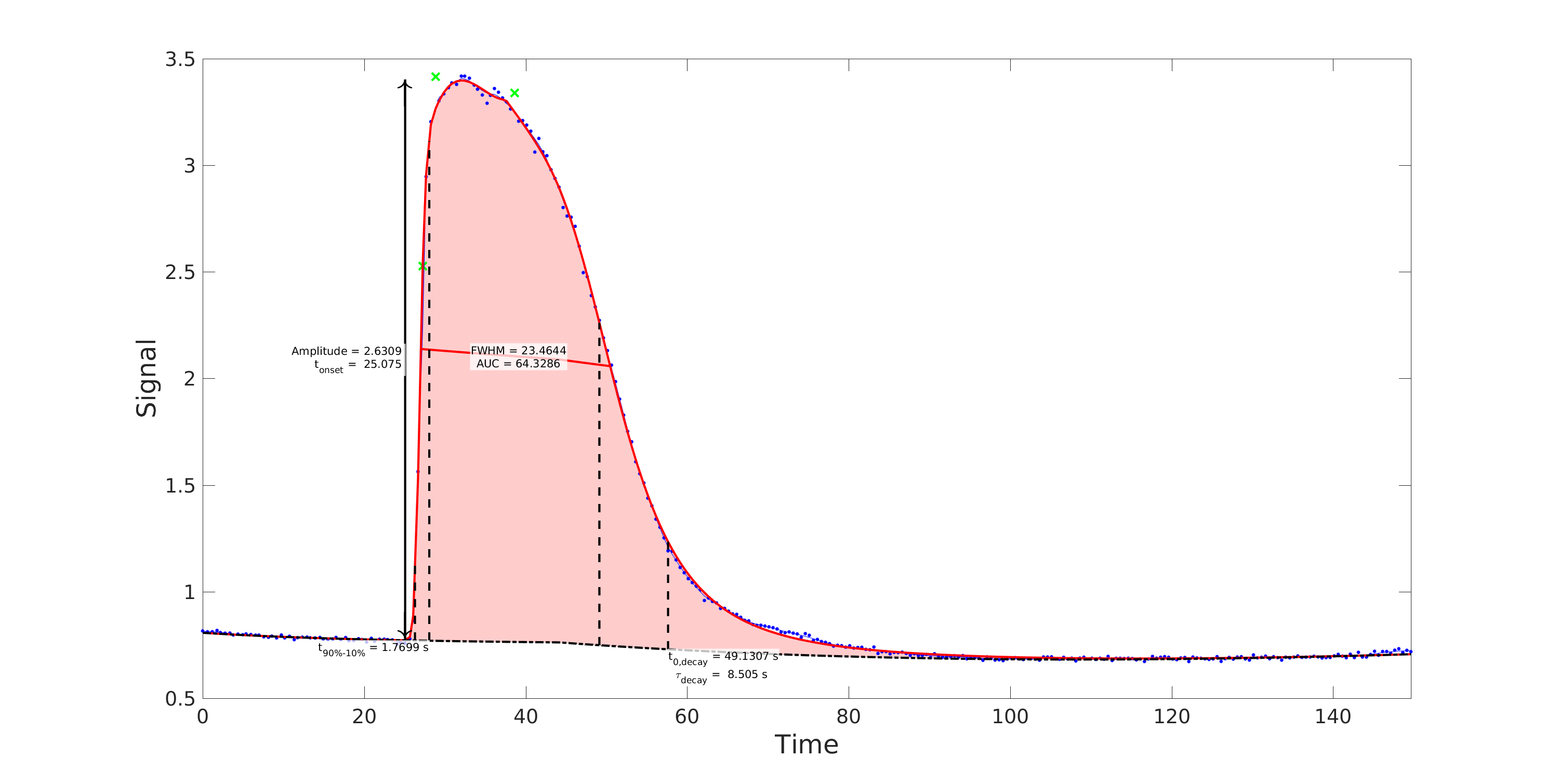

Supplement: Supplementary file 1 [file DataSheet1.ZIP › example/sample_outputs/example.Sheet_1.block_1.roi_1.png]

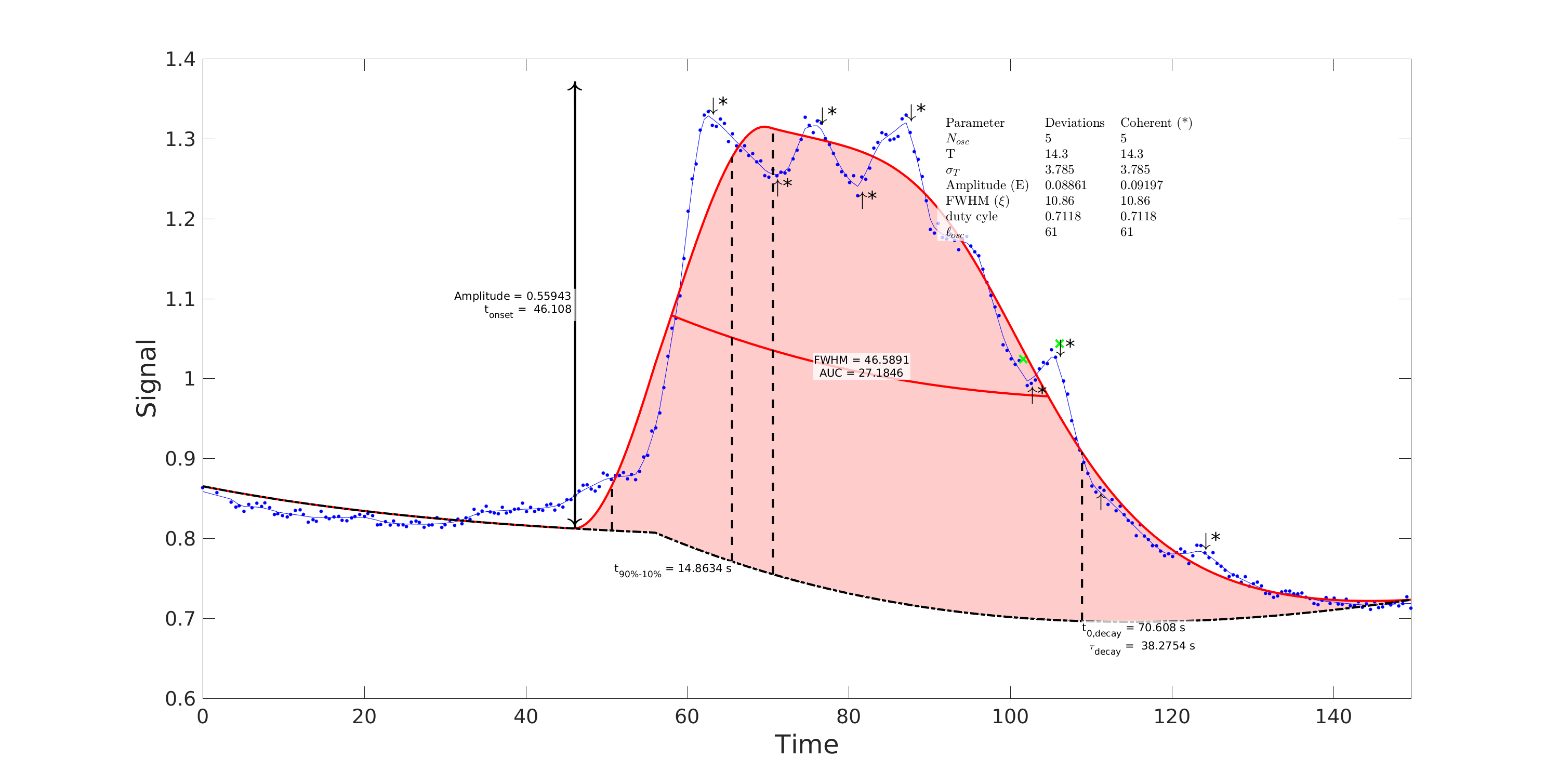

Supplement: Supplementary file 1 [file DataSheet1.ZIP › example/sample_outputs/example.Sheet_1.block_2.roi_1.png]

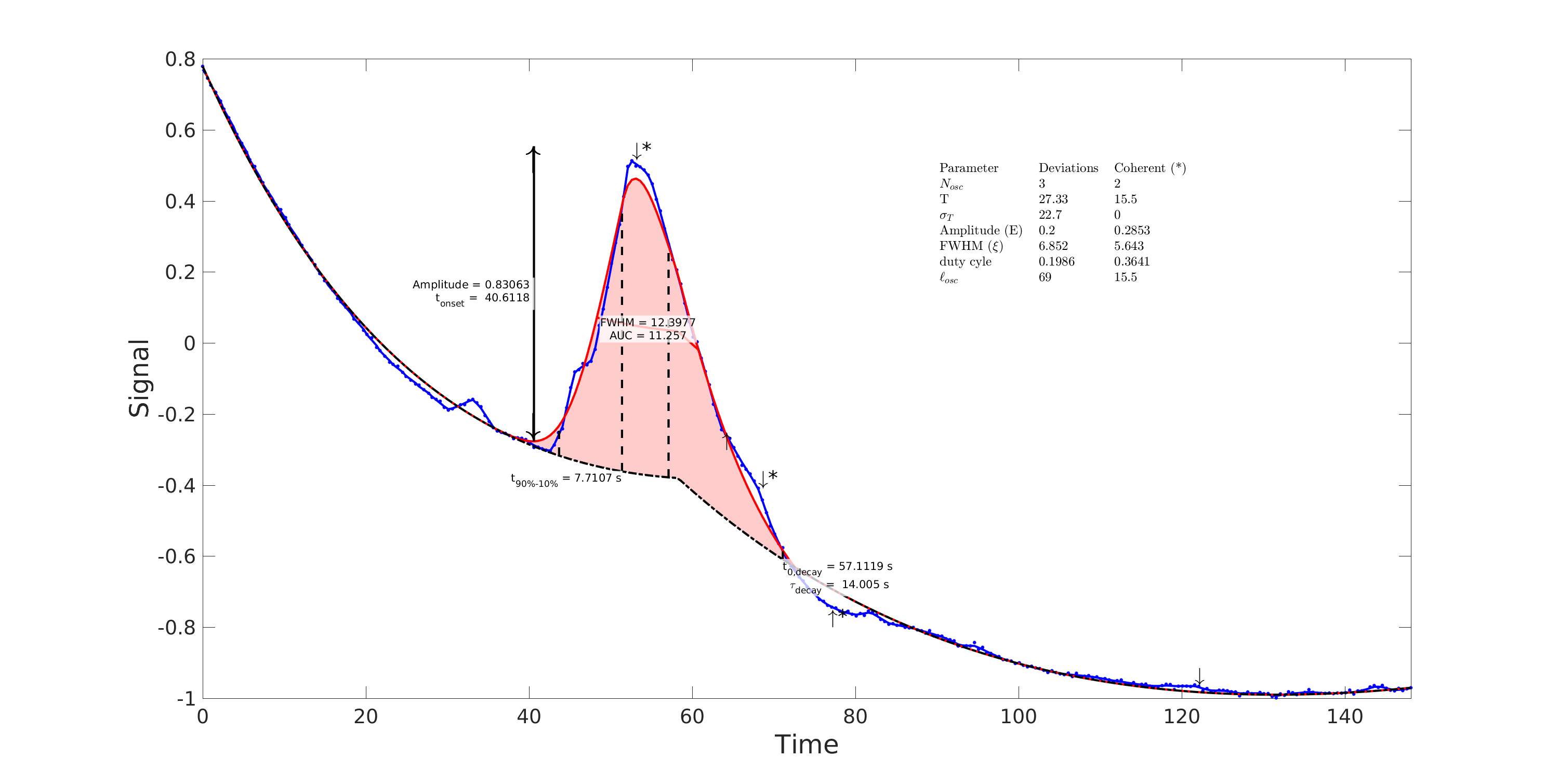

Supplement: Supplementary file 1 [file DataSheet1.ZIP › example/sample_outputs/example.Sheet_1.block_3.roi_1.png]

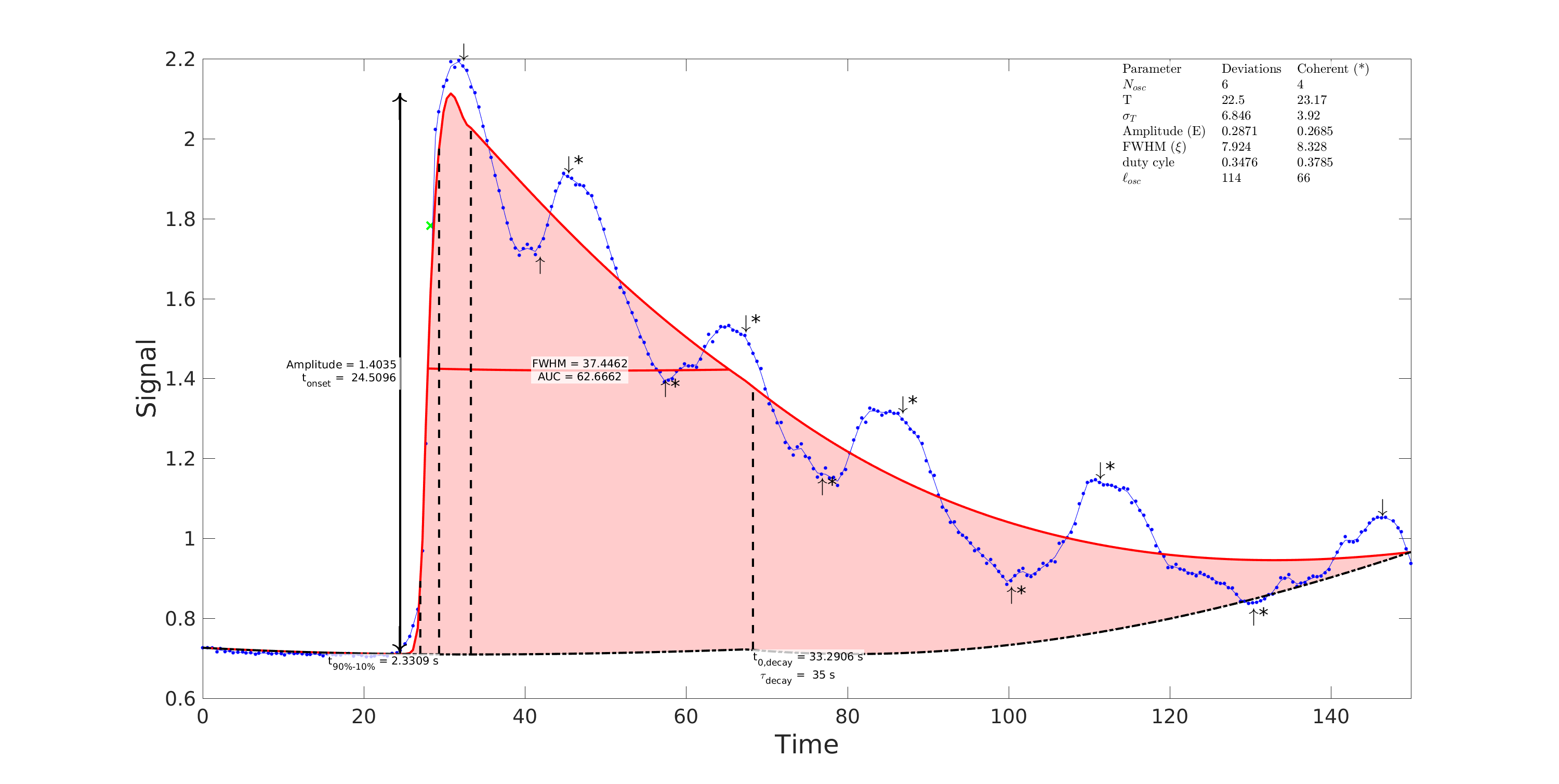

Supplement: Supplementary file 1 [file DataSheet1.ZIP › example/sample_outputs/example.Sheet_1.block_4.roi_1.png]

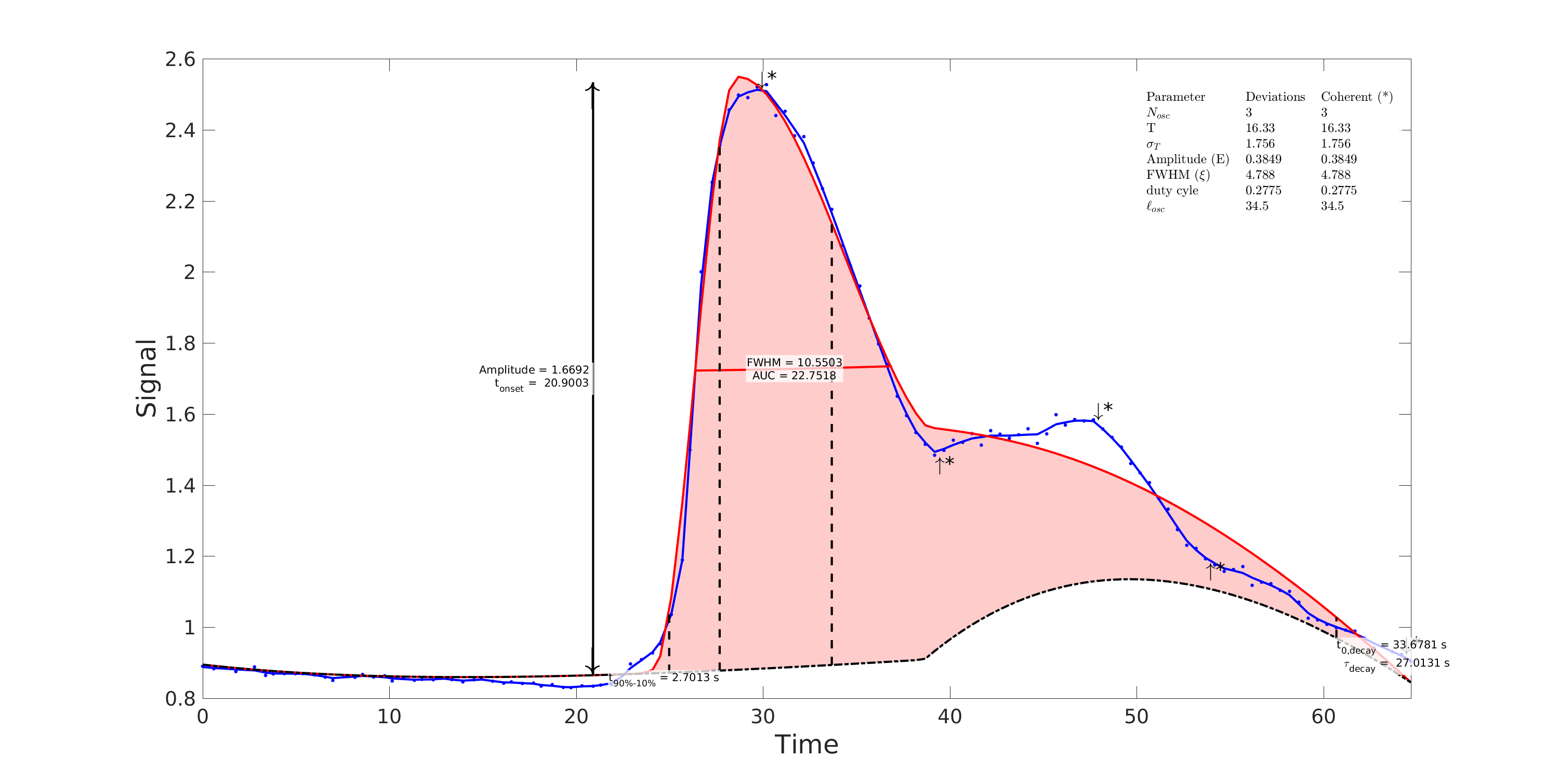

Supplement: Supplementary file 1 [file DataSheet1.ZIP › example/sample_outputs/example.Sheet_1.block_5.roi_1.png]
