# Supplementary material for: Systematic Characterization of Dynamic Parameters of Intracellular Calcium Signals
Source: Front Physiol. 2016 Nov 10;7:525. doi: 10.3389/fphys.2016.00525 (PMC5102910; doi:10.3389/fphys.2016.00525)
Supplement: Supplementary file 2 [file DataSheet2.docx]

# Appendix A

Two third-order Hermite polynomials have been used in a piecewise-defined manner to form a spline,

$$\begin{matrix} H_{1}\left(x\right)=\left(2x_{1}^{3}-3x_{1}^{2}+1\right) a_{0}+\left(x_{1}^{3}-2x_{1}^{2}+x_{1}\right) b_{0}+\left(-2x_{1}^{3}+3x_{1}^{2}\right) a_{1} \\ H_{2}\left(x\right)=\left(2x_{2}^{3}-3x_{2}^{2}+1\right) a_{1}+\left(-2x_{2}^{3}+3x_{2}^{2}\right) a_{2}+\left(x_{2}^{3}-x_{2}^{2}\right) b_{2}, \end{matrix}$$

where $x_{1}=\left(t-\rho_{act}\right)/\left(t_{de}-\rho_{act}\right)$ and $x_{2}=\left(t-t_{de}\right)/\left(\rho_{de}-t_{de}\right)$.The 6 parameters (the spline values) and $b_{0}$, $b_{1}$, and $b_{2}$ (the spline tangents) are completely defined in terms of the response parameters, as follows:

where implicit in this formalism that (to make $a_{1}$ correspond to the local maximum of the spline).
